# Interdisciplinary intervention (GAIN) vs. enhanced usual care for reducing severe post-concussion symptoms in adults 2–4 months post-injury: a stepped wedge cluster randomised controlled trial

**DOI:** 10.1016/j.eclinm.2026.104243

**Published:** 2026-09-22

**Authors:** Line Amalie Hellemose, Mille Møller Thastum, Henriette Holm Stabel, Lene Odgaard, Charlotte Ulrikka Rask, Noah D. Silverberg, Toril Skandsen, Hana Malá Rytter, Charlotte Nygaard, Peter Preben Eggertsen, Carsten B. Pedersen, Jørgen Feldbæk Nielsen

**Affiliations:** a Hammel Neurorehabilitation Centre and University Research Clinic, Aarhus University, Aarhus, Denmark; b Department of Child and Adolescent Psychiatry, Aarhus University Hospital, Aarhus N, Denmark; c Department of Clinical Medicine, Aarhus University, Aarhus N, Denmark; d Department of Psychology, University of British Columbia, Vancouver, BC, Canada; e Rehabilitation Research Program, Centre for Aging SMART, Vancouver Coastal Health Research Institute, Vancouver, BC, Canada; f Member, Djavad Mowafaghian Centre for Brain Health, Vancouver, BC, Canada; g Department of Neuromedicine and Movement Science, Norwegian University of Science and Technology (NTNU), Trondheim, Norway; h Clinic of Rehabilitation, St. Olav's Hospital, Trondheim University Hospital, Trondheim, Norway; i The Danish Concussion Center, Copenhagen, Denmark; j Department of Neurology, University Hospital Bispebjerg - Frederiksberg, Copenhagen, Denmark; k Department of Psychology, University of Copenhagen, Copenhagen, Denmark; l Department of Infectious Diseases, Aalborg University Hospital, Aalborg, Denmark; m National Centre for Register-Based Research, Aarhus University, Aarhus V, Denmark

**Keywords:** Brain concussion, Cognitive behavioural therapy, Graded exercise therapy, Illness perceptions, Intervention, Mild traumatic brain injury

## Abstract

**Background:**

Up to 30% of adults presenting to emergency departments develop persisting post-concussion symptoms (PPCS). Get going After concussIoN (GAIN), has previously shown promising effects in reducing PPCS in adolescents and young adults.

**Methods:**

This stepped wedge cluster randomised trial tested the effect of GAIN in reducing high levels of PPCS (i.e. a score ≥20 on the Rivermead Post-concussion Symptoms Questionnaire (RPQ)) 2–4 months post-injury in adults (18–60 years). GAIN was delivered in five clusters of municipalities in the Central Denmark Region sequentially allocated to implement the intervention, with one cluster transitioning to deliver GAIN every 3 months over six recruitment periods in the order of a computer-generated randomisation schedule. Participants were allocated to enhanced usual care (EUC) or GAIN + EUC depending on time and their place of residence. Masking of participants and therapists was not feasible. Primary outcome was change in RPQ total score from baseline to 3-month follow-up. Analyses were based on intention to treat using linear-mixed models. The trial is registered with ClinicalTrials.gov, NCT04798885.

**Findings:**

Between May 1, 2021 and November 1, 2022, 310 participants were included. The average difference in change in RPQ score between GAIN + EUC (n = 170) and EUC (n = 140) from baseline to 3-month follow-up was −2.4 points (95% CI −5.4 to 0.4, *p* = 0.09) in the adjusted model. GAIN reduced maladaptive, symptom-related perceptions and behaviours and patient satisfaction was high. GAIN + EUC was not associated with a greater improvement in psychological distress or physical and mental health than EUC. No serious adverse events were reported.

**Interpretation:**

This study could not confirm the hypothesis that GAIN added to EUC led to a larger long-term reduction in PPCS than EUC alone in adults. However, GAIN may improve long-term prognosis by reducing maladaptive, symptom-related perceptions and behaviours.

**Funding:**

The foundation Sygeforsikringen “danmark” and Central Denmark Region.

**Research in context**

**Evidence before this study**

Persisting post-concussion symptoms (PPCS) are common and associated with disability and higher use of social services and health care. Yet evidence for effective treatment is limited. Literature has demonstrated that PPCS are not fully explained by pathophysiological processes directly linked to the head-trauma but are best understood in terms of a bio-psycho-social illness model. Of notice, PPCS are associated with maladaptive, symptom-perpetuating cognitions and behaviours which may be amenable to treatment.

Based on a broad literature search (performed in 2013) on psychological and behavioural interventions for PPCS, we previously developed the intervention Get going After concussIoN (GAIN), an interdisciplinary, cognitive behavioural intervention that focus on modifying maladaptive cognitions and behaviours. We demonstrated in a previous trial that GAIN was effective in reducing PPCS in adolescents and young adults (15–30 years) when delivered in a specialised hospital setting.

In preparation for the present study, we searched PubMed and PsychINFO (January, 2020) for original research and review articles on psychological and behavioural interventions for PPCS published since the first GAIN trial, without language restrictions. Keywords were “brain concussion” (MeSH), or “concussion”, or “mild traumatic brain injury”, or “mTBI”, or “minor head injury”, or “post-concussion” in combination with “treatment”, or “intervention”, or “clinical trial” (MeSH), or “therapeutics” (MeSH). We repeated the search in January 2026.

Randomised controlled trials of interventions for PPCS were still limited and suffered from methodological weaknesses. In addition, due to heterogeneity in interventions and outcome measures, it is difficult to compare studies and perform meta-analysis. However, according to recent systematic reviews there is increasing evidence that interdisciplinary, cognitive behavioural interventions may be effective in reducing PPCS. Therefore, these interventions are still recommended both for patients with early-stage PCS (<3 months) and for patients who have developed more prolonged symptoms.

**Added value of this study**

The present study evaluated the effectiveness of GAIN in reducing PPCS in adults (18–60 years) when delivered by non-expert occupational therapists and physiotherapists in a municipality setting. We could not confirm the hypothesis that GAIN led to a larger long-term reduction in PPCS than enhanced usual care in adults. However, GAIN significantly reduced maladaptive, symptom-perpetuating cognitions and behaviours which may improve long-term prognosis, and patient satisfaction was high. In addition, we demonstrated that it was feasible to implement GAIN in a municipality setting, and to educate occupational therapists and physiotherapists to deliver the intervention as intended.

**Implications of all the available evidence**

Taken together, the existing literature suggests a possible effect of interdisciplinary, cognitive behavioural interventions for PPCS, but the null findings of the present study underscore the need for further high-quality studies. Future research should explore how to improve the long-lasting effect of GAIN on symptom severity and, in addition, the potential effect of GAIN on daily functioning.

Based on evidence that maladaptive cognitions and behaviours are key factors in the development of PPCS it may be argued that these are important intervention targets. The findings from the present study highlight the potential of GAIN to effectively modify maladaptive cognitions and behaviours.

## Introduction

Most people with concussion recover spontaneously within the first weeks after injury, but up to 30% of adults presenting to emergency departments or trauma centres develop persisting post-concussion symptoms (PPCS), defined as symptoms persisting for more than 3 months post-injury.^1^ PPCS include physical, emotional and cognitive symptoms, and is associated with prolonged disability, higher use of social services, higher health costs, and reduced labour market attachment.^2^^,^^3^ Still, evidence for effective treatment to prevent long-term sequelae after a concussion is sparse.^4^

Pathophysiological processes such as neurometabolic dysfunction and microstructural injury appear to not fully explain PPCS. The development of PPCS is likely influenced by various biological, psychological, social and environmental factors.^5^^,^^6^ Some may be predisposing, and others may be conceptualised as symptom perpetuating factors which may be modifiable.^7^

One such factor is patients' illness perceptions, which has been demonstrated to be associated with PPCS.^5^^,^^8^ Illness perceptions is a concept derived from Leventhal's Common Sense Model of Illness Representation. It may be defined as one's subjective understanding of symptoms and illness that serves as a guide for how to manage symptoms in everyday life in a subjectively meaningful way (i.e. illness behaviour).^8^ Examples of maladaptive illness perceptions associated with PPCS are pessimistic expectations for recovery and treatment, and catastrophic thoughts about symptoms. Such beliefs are associated with maladaptive illness behaviours such as fear-avoidance behaviour (i.e. avoiding activities that provoke symptoms out of fear that it will worsen the prognosis) and excessive rest, or the opposite, continuous overexertion, or so-called “all-or-nothing behaviour”, where the person is overdoing things when symptoms are abating and spending prolonged periods recovering when symptoms reappear.^5^^,^^9^ These findings regarding the role of illness perceptions in PPCS are in accordance with the influential predictive coding model of symptom perception, which is promising in terms of bridging the gap between psychological and neurobiological processes in PPCS.^10^ According to this model, PPCS may be understood as non-specific and related to a hypothesised, reversible disturbance in brain-body interaction and experience of bodily signals.^10^^,^^11^ In line with the above, interventions for PPCS that aim to modify maladaptive illness perceptions and associated behaviours seem promising.^4^^,^^12^^,^^13^ We previously developed Get Going After concussIoN (GAIN) as an intervention for adolescents and young adults (15–30 years) who still suffered from symptoms 2–6 months post-concussion. GAIN is based on a biopsychosocial model of PPCS. It is designed to interrupt the symptom perpetuating cycle and to reduce PPCS by modifying maladaptive illness perceptions and the associated behaviours drawing on elements from third-wave cognitive behavioural therapy. Patients are supported in gradual return to daily activities including physical activity.^9^ We tested the efficacy of the intervention in a randomised controlled trial (GAIN 1.0), where GAIN was delivered by an interdisciplinary team in a specialised hospital setting. We found that GAIN reduced the risk of still suffering from PPCS 3 and 12 months after end of intervention compared to enhanced usual care.^11^ However, the effect of GAIN when delivered in different settings, by different therapists, and to a broader age group remains to be evaluated.

The primary aim of the present study, GAIN 2.0, was to evaluate the effectiveness of GAIN added to enhanced usual care (EUC) when delivered by therapists in a municipality setting in reducing PPCS in adults (18–60 years) and preventing symptoms from becoming chronic. We employed a stepped wedge cluster randomised design which facilitated the gradual training of therapists and stepwise implementation of the intervention across the participating communities. We hypothesised that GAIN added to EUC would lead to larger reductions in PPCS at 3 months post-treatment compared with EUC alone.

## Methods

### Study design

GAIN 2.0 is a stepped wedge cluster randomised trial carried out in Central Denmark Region (1.4 million inhabitants) as a collaboration between Hammel Neurorehabilitation Centre and University Research Clinic and the municipalities. In total, 16 out of the 19 municipalities in Central Denmark Region had therapists that participated in the trial, but citizens from all municipalities were invited to participate. The municipalities are grouped into five healthcare-clusters collaborating with the primary healthcare system and general practitioners (GPs) in providing healthcare and rehabilitation to their citizens.

The five healthcare clusters were the unit of randomisation in GAIN 2.0. The aim of randomisation at this level was to reduce the risk that participants allocated to EUC might unintentionally receive treatment with elements from GAIN. Figure S1 (Supplemental Material) illustrates the stepped wedge cluster randomised design. There were six recruitment periods of each 3 months’ length.

A detailed description of the study protocol, including study design, outcomes, data collection, and assessment of potential participants, has been reported elsewhere.^14^

### Participants

From May 1, 2021, to November 1, 2022, participants were recruited (1) from a population-based cohort study, including all individuals diagnosed with concussion at one of the emergency departments (EDs) of Central Denmark Region, and (2) from general practitioners (GPs) who could refer patients with severe post-concussion symptoms 2–4 months after concussion. The rationale for this timeframe was that most patients will recover spontaneously within 1–2 months, and intervention in this period is futile and may cause iatrogenic harm. Patients from EDs were sent a questionnaire including the Rivermead Post-concussion symptoms Questionnaire (RPQ) 2 months after diagnosis. Patients with an RPQ ≥20 were identified as potential participants in GAIN 2.0 and invited to a clinical assessment to determine eligibility (cf. “Procedures”). Potential participants were informed that they would be allocated to either the EUC or GAIN intervention after inclusion.

Participants were included in GAIN 2.0 based on the following inclusion criteria: (1) concussion according to the diagnostic criteria recommended by the WHO Neurotrauma Task Force with the amendment that there must have been a direct contact between the head and an object in order to rule out pure acceleration-deceleration traumas^15^; (2) age 18–60 years at the time of the trauma; (3) moderate to high levels of post-concussion symptoms defined as a score of ≥20 points on the RPQ within one week of inclusion; (4) able to understand, speak and read Danish; and (5) resident in Central Denmark Region. There is no consensus for grading the severity of symptoms based on the RPQ. We defined treatment-demanding PPCS like in the GAIN 1.0 trial as a total score of ≥20 on the RPQ which exceeds typical symptom levels observed in the general population.^16^

Exclusion criteria were: (1) objective findings from a neurological examination indicating brain damage and/or self-reported neurosurgery in relation to the head trauma; (2) previous concussion within the last 2 years with ongoing PPCS at the time of the present concussion; (3) severe misuse of alcohol, prescription drugs and/or illegal drugs; (4) severe psychiatric co-morbidity that would impede participation in the intervention; and (5) inability to start the intervention within 7 months from concussion (the maximum time for eligibility (4 months) plus the maximum possible waiting time from enrolment to start of intervention (3 months)). The rationale for excluding individuals with ongoing PPCS from a previous concussion within the last 2 years was that GAIN is developed as an intervention to prevent the development of chronic symptoms. Severe psychiatric co-morbidity was defined as having received specialised psychiatric treatment within the last 2 years.

### Procedures

Patients from EDs and GPs were invited to a clinical assessment by a physician at Hammel Neurorehabilitation Centre to determine eligibility. The assessment included: (1) completion of a baseline questionnaire including the RPQ sent by email within one week prior to the clinical assessment; (2) a neurological examination following the Neurological Outcome Scale for Traumatic Brain Injury (NOS-TBI); and (3) a structured interview focussing on the patient's medical history and on evaluating the eligibility criteria. To ensure consistency in the verification of the concussion diagnosis, structured questions evaluating the key criteria for the diagnosis were incorporated into the interview based on previous recommendations.^17^ All patients received EUC after the assessment. Ineligible patients were guided to seek further advice from their GP if needed.

Under Danish and EU data protection legislation, information revealing racial or ethnic origin is classified as special category personal data, and collection of such data must be necessary and proportionate to the scientific aims of the study. As race and ethnicity were not prespecified as prognostic factors, effect modifiers, or stratification variables in the approved study protocol, this data was not collected.

### Enhanced usual care (EUC)

EUC was chosen as comparison group which aligns with the research question which was to determine whether GAIN improves concussion recovery over and above standard of care. The EUC was similar to the EUC in the GAIN 1.0 trial, and more comprehensive than usual care of patients with PPCS.^9^^,^^11^ It was provided individually and in-person to all patients by an occupational therapist immediately after the clinical assessment. They received 30 min of psychoeducation about the bio-psycho-social understanding of PPCS, including reassurance that the symptoms, although unpleasant, are not signs of anything dangerous that should be avoided, and advice on adaptive illness behaviours. The psychoeducation provided in EUC was in accordance with the psychoeducation provided in the GAIN intervention. Neither the occupational therapist delivering EUC nor the physicians performing the clinical assessment were involved in providing the GAIN intervention.

### Get going after concussIoN (GAIN)

The GAIN intervention provided was the same as in GAIN 1.0.^9^^,^^11^ Table S1 (Supplemental Materials) displays the over-all structure of the 8-week interdisciplinary intervention, which is described in detail in the study protocol.^14^ The group sessions followed a fixed structure supported by a slide presentation and a written manual, and the individual sessions followed a semi structured manual. The GAIN manuals and materials are available online in English at this link: https://www.fagperson.hospitalsenhedmidt.dk/afdelinger/regionshospitalet-hammel-neurocenter/university-research-clinic/forskning-om-hjernerystelse/gain/.

The primary objective of GAIN was to indirectly reduce PPCS by changing maladaptive, symptom-related perceptions and behaviours, which were the hypothesised mechanisms of change.^5^^,^^7^^,^^8^ Physical activity was an important treatment target, addressed in all participants. Participants were encouraged to be physically active every day with any aerobic activity of their choice (i.e. walking, running, biking, cleaning, gardening) beginning at an individualised starting level regarding intensity and duration and only gradually increase from there as tolerated. A temporary worsening of symptoms was acceptable if it subsided by the next day at the latest.

### Delivery of GAIN

Nine physiotherapists, eight occupational therapists, and two experienced neuropsychologists with specialised knowledge of PPCS and GAIN, and with training in third-wave cognitive behavioural therapy, were engaged in delivering GAIN in one or two designated locations within each of the five health-care clusters in interdisciplinary teams of three therapists. About 2 months before a health-care cluster transitioned to delivering GAIN, the therapists from the cluster completed a two-day training course in GAIN led by the neuropsychologist and the physiotherapist who had been engaged in the development of the original GAIN programme. During each GAIN course, the interdisciplinary team had a weekly, 1-h virtual meeting (1) to facilitate interdisciplinary knowledge sharing, cooperation, and coordination, and (2) to provide the therapists with supervision from the neuropsychologist to ensure that the GAIN intervention was delivered as intended. All treatment sessions were audio recorded. An external occupational therapist, with knowledge about GAIN from completing the two-day training course in the intervention, listened to a random sample of 10% of the individual sessions to evaluate if the therapists adhered to the GAIN manual according to a predefined list of treatment elements.

### Randomisation and masking

Participants were allocated to GAIN + EUC or EUC depending on calendar time and their place of residence (i.e. which health-cluster). Health-care clusters (i.e. the unit of randomisation) were sequentially allocated to implement the intervention with one cluster transitioning to deliver GAIN every 3 months in the order of randomisation (Figure S1, Supplemental Materials). A randomisation schedule prepared by a statistician uninvolved in other trial procedures determined the sequence. The randomisation schedule was safely stored and only accessible to the statistician. Three months before a cluster transitioned, the statistician informed the project coordinator, who subsequently notified key personnel in the cluster and the study's data manager. Within 2 weeks from the clinical assessment the patient received an auto-generated email with information on allocation depending on their place of residence. The physicians assessing for eligibility and the therapist providing EUC were blinded to allocation of health-clusters. Due to the nature of the intervention, masking of participants and therapists was not feasible. Due to the study design, masking of the analytic statistician was not feasible. The data manager was not involved in the data analysis.

### Ethics

“The study was conducted in accordance with the Declaration of Helsinki and the principles of Good Clinical Practice. The trial was listed at the record of processing activities for research projects including the General Data Protection Regulation in Central Denmark Region (no. 1-16-02-69-21) and approved by the Committee of Health Research Ethics of Central Denmark Region (no. 1-10-72-330-20). The trial was prospectively registered at ClinicalTrials.gov (NCT04798885) on March 12, 2021, prior to enrolment of the first participant.

Participants were included after written informed consent at the individual level based on written information on the GAIN 2.0 study, which was emailed to potential participants, and oral information provided by a medical doctor through a telephone consultation within a week before the clinical assessment.

### Outcomes

Electronic questionnaires were distributed to each patient at baseline (T0, within one week before clinical assessment), at the end of intervention (T1), at 3-month follow-up (T2, primary endpoint), and at 12-month follow-up (T3). Table S2 (Supplemental Materials) shows an overview of outcome measures and measurement points.

### Primary outcome

Primary outcome was mean change in post-concussion symptoms level as measured by the RPQ total score (16 items, range 0–64) from baseline to 3 months follow-up.^18^ A higher score indicates a more severe symptom-level. In addition to the total score, somatic (9 items, range 0–36), cognitive (3 items, range 0–12), and emotional (4 items, range 0–16) subscores were calculated, based on evidence that the RPQ is more likely to have a three-factor structure than a one-factor structure.^16^

### Treatment targets

The hypothesised mechanisms of change were symptom-related illness perceptions and behaviours. Symptom-related perceptions were measured by The Brief Illness Perception Questionnaire (B-IPQ).^19^ Symptom-related behaviours were measured by two subscales of The Behavioural Response to Illness Questionnaire (BRIQ): “limiting behaviour”, corresponding to excessive rest, and “all-or-nothing behaviour”.^20^ Furthermore, we applied a single-item measure of fear avoidance behaviour. Participants were asked how strongly they agreed with the following statement “I avoid activities that might make my symptoms worse” over the past week, on a 10-point Likert scale from 1 (Strongly disagree) to 10 (Strongly agree). The sum score of the B-IPQ and the separate sum scores of the two subscales from BRIQ were each transformed into scales between 0 (best) and 100 (worst), and the Fear Avoidance measure was transformed into a scale between 10 (best) −100 (worst) for more uniform graphic presentation of the results.

### Secondary outcomes

Physical and mental health was measured by the Physical and Mental Component Summaries from The Short Form 36 Health Survey (SF 36), 2nd version, which were converted into T-scores based on US normative data. Illness worry was measured by Whiteley-6 sum score (range 0–24).^21^ Psychological distress was measured by the sum score on the Symptom Checklist-8 (SCL-8, range 0–32), a subscale derived from the Symptom Checklist-90-R.^22^

### Additional measures

Subjective improvement in general health was measured in the GAIN + EUC group by The Clinical Global Impression Scale. The participants were asked: “Compared to before you started the treatment, how would you describe your health today?” Answers were rated between “1” (“much worse”) and “5” (“much better”).

Patient satisfaction was measured in the GAIN + EUC group by The Experience of Service Questionnaire (ESQ) evaluating satisfaction with the care and the environment. Items were rated “Certainly true”, “Partly true”, Not true or “Don't know” and responses were evaluated descriptively.

Adverse events were monitored by asking the participants the following open-ended question to be answered in free text: “If you have in any way experienced that your condition has worsened by participating in the treatment project, you can describe it here”.

### Statistical analysis

The sample size calculation was performed using RPQ data from GAIN 1.0. In GAIN 1.0, we found a mean difference in the improvement of 7.6 points between GAIN + EUC and EUC on the total RPQ score 3 months after end of intervention. In GAIN 2.0, we hypothesised a difference in the improvement of 7 points between groups on the RPQ. Using the data from GAIN 1.0, the statistical power of the stepped-wedged design was estimated. With the assumptions of a population mean RPQ change of 9.3 points of GAIN + EUC compared with EUC, a measurement variance of 137, a variance between clusters of 0.97, a statistical significance level of 5%, and a statistical power of 80%, we estimated that 180 participants were needed to reach a statistical power of 80%.^14^ As a result of the pre-specified stepped-wedge design, it was not possible to ensure an equal distribution of participants to GAIN + EUC and EUC, respectively.

Analyses were handled on an intention-to-treat basis. Time specific RPQ scores were analysed using a restricted maximum likelihood (REML) based on repeated measures approach. The linear mixed model includes the fixed, categorical effects of recruitment period (6 levels), treatment (GAIN + EUC vs. EUC) and time (baseline, end of intervention, 3-month follow-up, and 12-month follow-up) and treatment by time interaction. The model included a random intercept of cluster and a random intercept of participants (within clusters). However, as this model failed to converge, and the model checking offered by Hemmings et al. identified unspecific secular trends between clusters, the random part of the linear mixed model was extended to control for interaction between site and calendar time.^23^ The linear mixed model uses all available outcome data from participants with incomplete follow-up and provides unbiased estimation under missing completely at random or missing at random assumptions, provided that the model is correctly specified.^24^^,^^25^

The target measures and secondary outcomes were analysed using linear mixed models similar to those described for the primary outcome. However, for the secondary outcomes the analysis was performed by using information from only two time points (these measures were only administered at baseline and 3-month follow-up). All outcomes were analysed in both crude models and in models adjusting for predefined potential confounders, i.e. prognostically important baseline characteristics. The basic adjustment included age, sex, type of recruitment (ED/referred from GP), and previous mental health status (i.e. previous psychiatric diagnosis at any timepoint in life prior to the concussion, yes/no). The full adjustment added educational level, functional somatic syndrome diagnosis prior to injury (yes/no), and current psychological distress (SCL-8).^22^ We did not include chronic medical conditions prior to injury in the adjustment because it was a broad category including a wide range of heterogeneous medical conditions (Table 1) which to our knowledge are not known risk factors for developing PPCS. In a post-hoc analysis, the treatment-specific effect subdivided by all the potential confounders was evaluated. Additionally, per-protocol analysis for completers (defined as participants who received one or more group sessions and at least one individual session) was performed to provide an estimate of the efficacy of GAIN among those who completed the intervention as planned. Data from the ESQ on patient satisfaction was analysed descriptively.

**Table 1:** Baseline characteristics of the intention-to-treat population (n = 310).

	GAIN + EUC (n = 170)	EUC (n = 140)
Age	37.6 (12.6)	37.3 (12.7)
Sex (female)	123 (72%)	97 (69%)
Education level		
Basic education (7–11 years)	19 (11%)	15 (11%)
High school (13 years)	25 (15%)	23 (16%)
Vocational education	27 (16%)	25 (18%)
Higher education	95 (56%)	70 (50%)
Other (*incl. missing*)	4 (2%)	7 (5%)
Workability prior to concussion (self-reported)^a^		
“Best of lifetime” (workability score = 10)	105 (62%)	83 (59%)
Less than ”best of lifetime” (workability score <10)	63 (37%)	49 (35%)
*Missing*	2 (1%)	8 (6%)
Current work status		
No sick leave	37 (22%)	24 (17%)
Part time sick leave	49 (29%)	46 (33%)
Full time sick leave	82 (48%)	68 (49%)
*Missing*	2 (1%)	2 (1%)
Cohabitant status		
Living alone	27 (16%)	23 (16%)
Living with parents or partner	118 (69%)	96 (69%)
Living with friends, roommates, or siblings	11 (6%)	8 (6%)
Living with other	14 (8%)	5 (4%)
*Missing*	0 (0%)	8 (6%)
Recruitment		
Emergency department	59 (35%)	52 (37%)
General practitioners	111 (65%)	88 (63%)
Clusters		
Cluster 1 (Vest)	42 (25%)	9 (6%)
Cluster 2 (Midt)	41 (24%)	22 (16%)
Cluster 3 (Aarhus)	58 (34%)	54 (39%)
Cluster 4 (Randers)	25 (15%)	27 (19%)
Cluster 5 (Horsens)	4 (2%)	28 (20%)
Mechanism of injury		
Traffic accident, driver	15 (9%)	13 (9%)
Traffic accident, pedestrian or cyclist	9 (5%)	11 (8%)
Fall	50 (29%)	53 (38%)
Direct blow to the head (not violence)	69 (41%)	52 (37%)
Violence/assault	5 (3%)	3 (2%)
Other	22 (13%)	8 (6%)
Time since injury, months	4.6 (1.2)	4.8 (1.2)
Mental Health, previous (self-reported^b^)		
No previous psychiatric diagnosis	99 (58%)	101 (72%)
Depression	28 (16%)	16 (11%)
Anxiety	46 (27%)	25 (18%)
Other^c^	20 (12%)	16 (11%)
Current mental health issues		
SF-36 mental health, T-score (mean/sd)	37.1 (9.0)	36.9 (10.2)
*Missing*	14 (8%)	11 (8%)
Psychological distress, SCL-8	10.3 (6.5)	10.0 (6.7)
*Missing*	0 (0%)	1 (<1%)
Physical health (self-reported^b^)		
Chronic somatic condition diagnosed prior to injury^d^	86 (51%)	51 (36%)
Functional somatic syndrome diagnosed prior to injury^e^	50 (29%)	26 (19%)
SF-36 physical health, T-score (mean/sd)	40.9 (7.0)	40.6 (7.3)
*Missing*	14 (8%)	11 (8%)
Prior concussion(s)		
No previous concussions	101 (59%)	52 (37%)
1-2 previous concussions	50 (29%)	35 (25%)
More than 3 previous concussions	19 (11%)	8 (6%)
*Missing*	0 (0%)	45 (32%)^g^
Post-concussion symptoms		
RPQ (0–64) (mean/sd)	36.5 (9.3)	36.6 (10.1)
RPQ somatic factor (0–36) (mean/sd)	19.1 (5.6)	19.0 (6.2)
RPQ cognitive factor (0–12) (mean/sd)	8.1 (3.1)	8.6 (2.7)
RPQ emotional factor (0–16) (mean/sd)	9.3 (3.4)	9.1 (3.9)
Illness worry		
Whiteley-6 (mean/sd)	4.5 (4.4)	5.1 (5.2)
*Missing*	0 (0%)	1 (<1%)
Illness perceptions and illness behaviours		
BRIQ “all or nothing” behaviour” (0–100)	47.4 (16)	46.8 (17.9)
*Missing*	0 (0%)	3 (2%)
BRIQ “limiting behaviour” (0–100)	65.6 (18.0)	66.2 (18.2)
*Missing*	0 (0%)	3 (2%)
B-IPQ (0–100)	61.4 (9.4)	58.8 (11.1)
*Missing*	1 (<1%)	2 (2%)
FAB ”Fear Avoidance Behaviour” (10–100)	69.7 (23.0)	67.8 (23.4)
*Missing*	9 (5%)	80 (57%)^g^
Expectancy for treatment-effect (0–100)^f^	63.2 (22.6)	69.3 (22.8)
*Missing*	1 (<1%)	2 (1%)

Data are n (%), mean (SD), if not stated otherwise.

Abbreviations: B-IPQ = Brief Illness Perception Questionnaire; BRIQ = Behavioural Response to Illness Quetionnarie; EUC = Enhanced Usual Care; GAIN = Get going After concussIoN; RPQ = Rivermead Post-concussion Symptoms Questionnaire; SCL-8 = Symptom Checklist-8; SF-36 = Short Form 36 Health Survey.

a Patients were asked: “How would you score your workability prior to the concussion on a scale from 0 to 10, where 0 is unable to work, and 10 is “best of lifetime” work ability? If you went to school or were in training, consider it your work."

b Patients were asked: “Have a physician at any timepoint in your life prior to the concussion given you a diagnosis of …” (yes/no).

c “Other” includes: ADHD, Autism, Bipolar disorder, OCD, Psychosis or Schizophrenia, Eating disorder, Personality disorder.

d Chronic somatic disorders include: Cancer, Diabetes, Hypertension, Heart disease, Stroke, AMI (Acute Myocardial Infarction), Neurological disorder, Asthma, Psoriasis, Osteoarthritis, Rheumatoid arthritis, COPD (Chronic Obstructive Pulmonary Disease), Osteoporosis.

e Functional somatic syndromes include: Whiplash, Irritable bowel syndrome, Chronic fatigue syndrome, Fibromyalgia.

f Patients were asked: “How likely do you think it is that you will fully recover (0 = not likely, 10 = very likely)?"

g We did not ask about previous concussions and fear-avoidance behavior from the beginning of the recruitment, where we exclusively or mostly recruited patients to the EUC group (1st and 2nd period). Information about previous concussions was collected from 1 August, 2021. The FAB measure was applied from 14 October, 2021.

R version 3.1 was used for data-curation and statistical analyses. The statistical significance level was set to 0.05.

### Role of the funding source

The project was funded by Sygeforsikringen “danmark”. Central Denmark Region (an administrative region of Denmark with responsibility of public health care) provided financial support to the outpatient clinic at Hammel Neurorehabilitation Centre where potential participants were seen for clinical assessment. The funders were not involved in the study design, data collection, data analysis, or data interpretation, or in writing the report.

## Results

Between May 1, 2021 and November 1, 2022, 310 participants were included in the study. In total, 170 participants were allocated to GAIN + EUC (23 treatment groups) and 140 participants to EUC. Due to the predefined stepped-wedge design, all potential participants were invited to assessment throughout the study period, and thus more participants than estimated by the power calculation (n = 180) were included. Fig. 1 shows the flowchart and the timeline of assessment. Follow-up data from the last patient was collected on 1 June, 2023. Loss to 3-month follow-up was slightly larger in the EUC group (56/140, 40%) than in the GAIN + EUC group (51/170, 30%). In total, 29 patients (17%) dropped out (21 patients before and eight patients after intervention start); the median number of group sessions for completers were 3 (Inter Quartile Range (IQR) 3–2) and the median number of individual sessions were 6 (IQR 6–4).

**Fig. 1:**
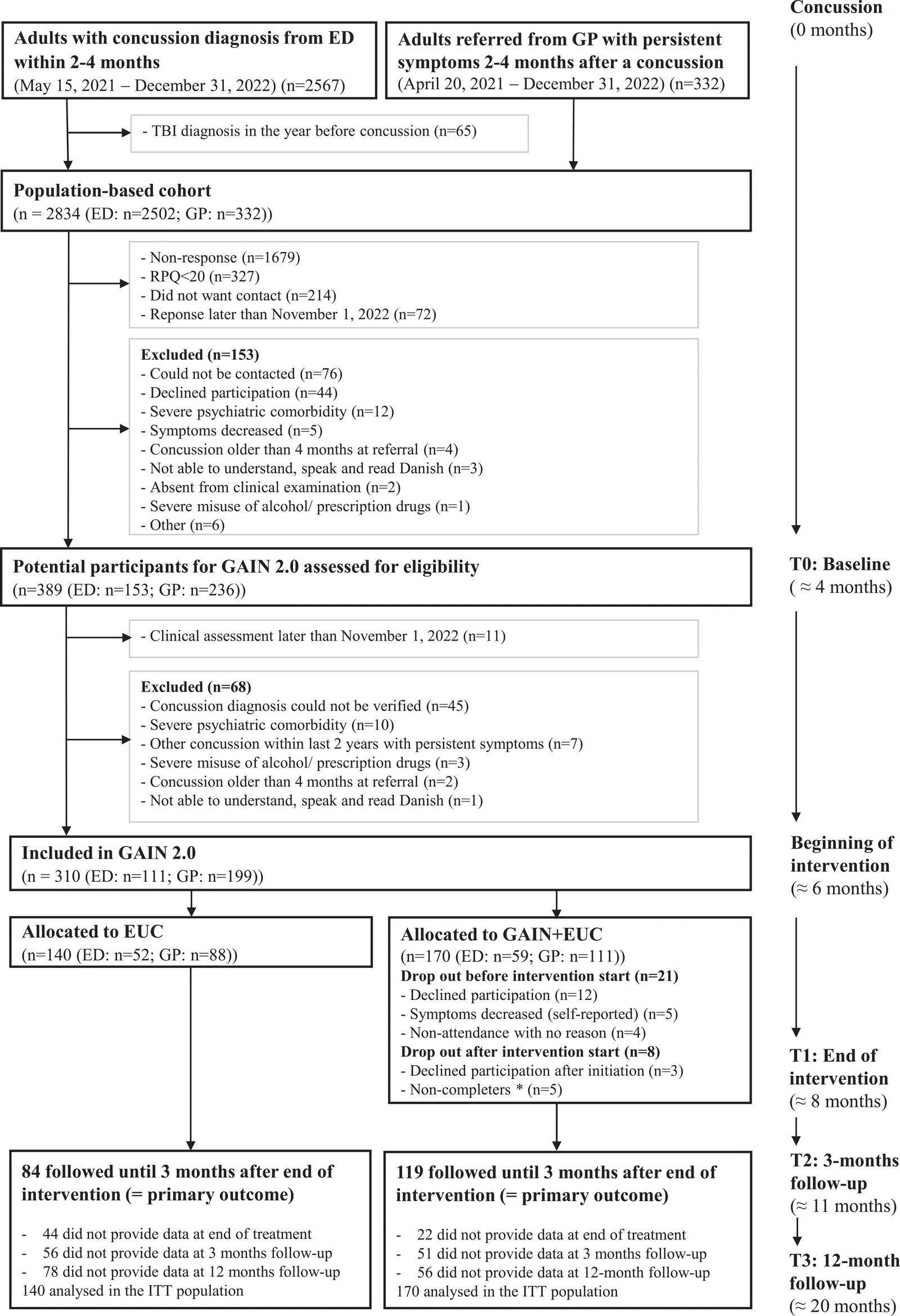
CONSORT Flowchart and timeline. The timeline represents approx. months after concussion according to the measurement timepoints defined in the trial protocol. ∗Non-completers: completed less than one group session and one individual session (i.e. consented to participate, but were not able to receive the intervention per-protocol due to practical or personal challenges). Abbreviations: ED = Emergency Department; EUC = Enhanced Usual Care; GP = General Practitioner; RPQ = Rivermead Post-concussion symptoms Questionnaire. TBI: Traumatic brain injury.

Table 1 shows the baseline characteristics of the included participants according to allocated intervention arm. Participants allocated to GAIN + EUC and EUC were demographically and clinically similar at baseline, except that a larger proportion in the GAIN + EUC group than in the EUC group had prior to the concussion been diagnosed with a psychiatric disorder, a functional somatic syndrome, or a chronic medical condition.

The review of the random sample of audio recordings from the individual sessions confirmed that the therapists overall adhered to the treatment strategies. However, there was a low adherence to treatment strategies derived from third-wave cognitive behavioural therapy targeting maladaptive symptom-perpetuating perceptions (applied in 57% of sessions) such as exploring the relationship between thoughts and behaviour (Table S3, Supplemental Materials).

### Primary outcome

Overall, the mean RPQ total score decreased by time since concussion (Fig. 2). Table 2 shows unadjusted and adjusted average treatment effect from baseline to 3-month follow-up. At 3-month follow-up, the difference in change between treatment groups in the RPQ total score favoured GAIN + EUC but the confidence interval included the possibility of no difference across all adjustment levels. The average difference in change was −2.3 (95% CI −5.2 to 0.6, *p* = 0.11) in the unadjusted model, and −2.4 (95% CI −5.4 to 0.4, *p* = 0.09) in the fully adjusted model. In the following, we refer to the fully adjusted model unless mentioned otherwise. Of note, at end of intervention the difference in change between treatment groups substantially favoured GAIN + EUC across all adjustment levels (e.g. −5.5 (95% CI −8.3 to −2.9, *p* < 0.001) in the fully adjusted model), but this difference attenuated at 3-month and at 12-month follow-up. The RPQ subscale analysis (Table 2) revealed similar findings, as well as the per-protocol analysis (Table S4, Supplemental Materials), although the difference in change in the fully adjusted per-protocol analysis at 3-month follow-up was larger than in the primary analysis (−2.7 (95% CI −5.7 to 0.01, *p* = 0.05).

**Fig. 2:**
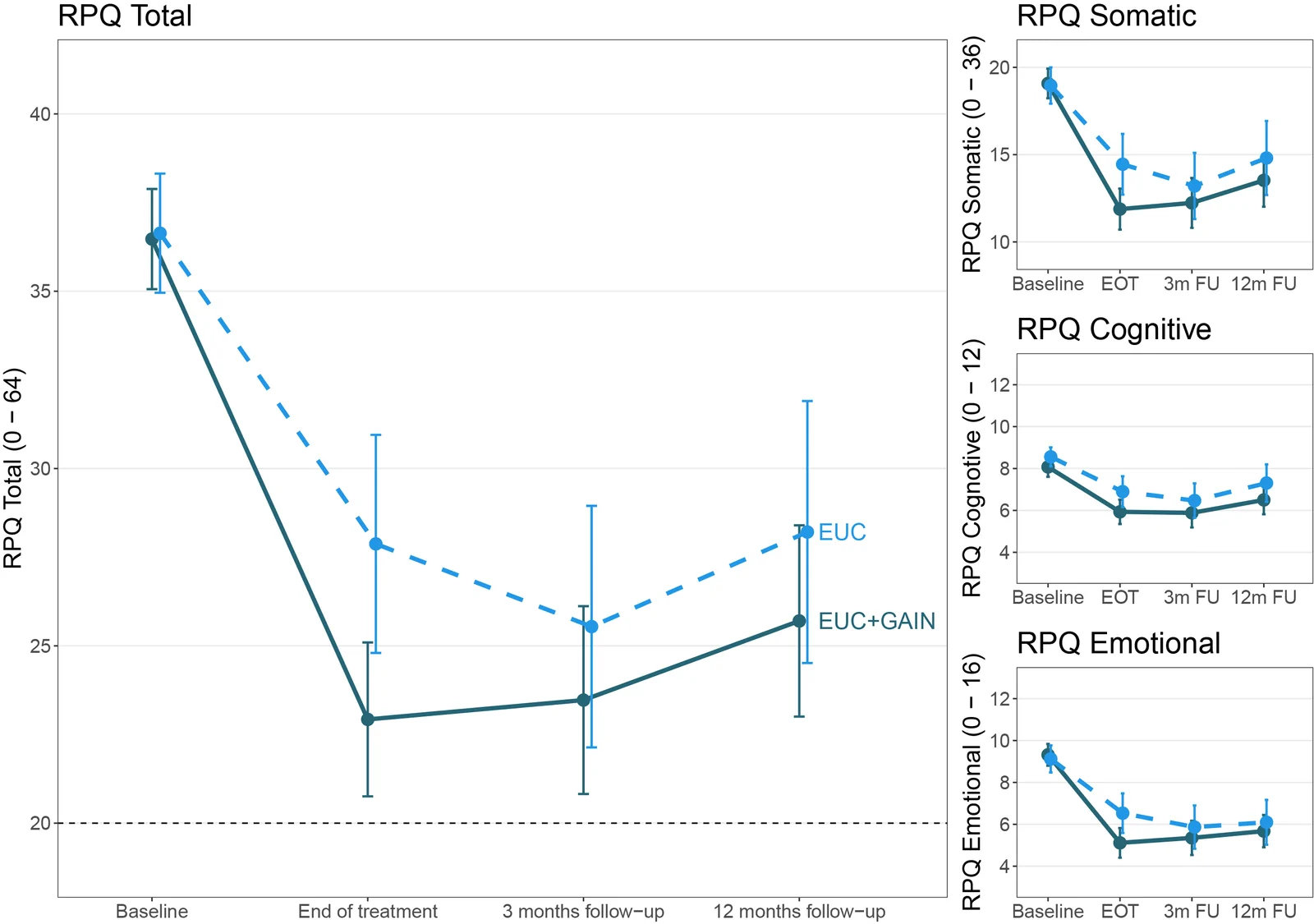
Unadjusted estimates of the mean Rivermead Post-Concussion Symptoms Questionnaire (RPQ) total score are presented for the GAIN + EUC and EUC groups at baseline, end of treatment (EOT), 3-month follow-up (FU), and 12-month follow-up. Additionally, estimates for the somatic, cognitive, and emotional subscales of the RPQ are shown for the same time points. The blue dotted line represents EUC and the dark solid line represents EUC + GAIN. Errorbars are 95% confidence intervals.

**Table 2:** Difference in change between GAIN + EUC and EUC in RPQ total score and its subscales from baseline to end of treatment, 3-month follow-up, and 12- month follow-up (n = 310).

Outcome	Unadjusted			Basic adjustment^a^			Full adjustment^b^		
	Mean	95% CI	*p*	Mean	95% CI	*p*	Mean	95% CI	*p*
RPQ, total (0–64)									
End of treatment	−5.30	(−8.05; −2.62)	<0.001	−5.36	(−8.17; −2.74)	<0.001	−5.49	(−8.27; −2.91)	<0.001
3-months follow-up	−2.29	(−5.24; 0.57)	0.11	−2.43	(−5.44; 0.37)	0.09	−2.40	(−5.36; 0.37)	0.09
12-months follow-up	−1.40	(−4.54; 1.72)	0.38	−1.45	(−4.66; 1.59)	0.34	−1.58	(−4.77; 1.42)	0.29
RPQ, somatic subscale (0–30)									
End of treatment	−3.02	(−4.51; −1.55)	<0.001	−3.04	(−4.55; −1.60)	<0.001	−3.11	(−4.61; −1.71)	<0.001
3-months follow-up	−1.34	(−2.93; 0.22)	0.09	−1.40	(−3.02; 0.14)	0.07	−1.39	(−2.99; 0.13)	0.072
12-months follow-up	−0.80	(−2.51; 0.89)	0.35	−0.82	(−2.56; 0.85)	0.32	−0.88	(−2.60; 0.76)	0.29
RPQ, cognitive subscale (0–12)									
End of treatment	−0.48	(−1.29; 0.26)	0.19	−0.52	(−1.36; 0.19)	0.14	−0.54	(−1.38; 0.16)	0.12
3-months follow-up	−0.28	(−1.15; 0.51)	0.44	−0.34	(−1.23; 0.42)	0.34	−0.33	(−1.23; 0.43)	0.34
12-months follow-up	−0.15	(−1.08; 0.72)	0.69	−0.20	(−1.15; 0.64)	0.58	−0.21	(−1.17; 0.62)	0.55
RPQ, emotional subscale (0–16)									
End of treatment	−1.84	(−2.80; −0.89)	<0.001	−1.90	(−2.87; −0.97)	<0.001	−1.90	(−2.84; −0.98)	<0.001
3-months follow-up	−0.72	(−1.75; 0.29)	0.16	−0.80	(−1.85; 0.19)	0.11	−0.76	(−1.77; 0.23)	0.13
12-months follow-up	−0.50	(−1.60; 0.61)	0.38	−0.55	(−1.67; 0.54)	0.31	−0.60	(−1.69; 0.47)	0.27

Average treatment effect on the RPQ (primary outcome measure) and RPQ subscales (post-hoc analysis) based on an unadjusted and adjusted linear mixed model.

Abbreviation: RPQ = Rivermead Post-concussion symptoms Questionnaire.

a Basic adjustment: Adjusted for age, sex, recruitment (ED/referred from GP), and previous mental health status (i.e. previous psychiatric diagnosis, yes/no).

b Full adjustment: Adjusted for basic adjustments and education level, functional somatic syndrome diagnosis prior to injury (yes/no), current psychological distress (SCL-8).

A post-hoc analysis revealed that the average treatment effect at 3-month follow-up varied substantially across participants with and without a psychiatric diagnosis at any timepoint in life prior to the concussion (*p* = 0.02), but the average treatment effect did not vary substantially by age, sex, calendar period, cluster, education, previous functional disorder, and current psychological distress (Table S5, Supplemental Materials).

### Treatment targets and secondary outcomes

Table 3 shows unadjusted and adjusted average treatment effect from baseline to 3-month follow-up on treatment targets and secondary outcomes.

**Table 3:** Difference in change in treatment targets and secondary outcome measures between GAIN + EUC and EUC from baseline to 3-month follow-up (n = 310).

Outcome	Unadjusted			Basic adjustment^c^			Full adjustment^d^		
	Mean	95% CI	*p*	Mean	95% CI	*p*	Mean	95% CI	*p*
Treatment targets									
B-IPQ^a^	−10.20	(−14.76; −5.32)	<0.001	−10.40	(−14.99; −5.55)	<0.001	−10.29	(−14.79; −5.37)	<0.001
BRIQ-lim^a^	−5.93	(−11.53; −0.33)	0.04	−6.01	(−11.60; −0.41)	0.04	−5.90	(−11.50; −0.28)	0.04
BRIQ-all^a^	−7.61	(−12.64; −2.42)	0.004	−7.74	(−12.83; −2.59)	0.003	−7.76	(−12.84; −2.66)	0.003
Secondary outcomes									
SCL-8	0.27	(−1.53; 2.10)	0.76	0.01	(−1.84; 1.77)	0.97	0.06	(−1.78; 1.83)	0.98
Whiteley-6	−1.42	(−2.76; −0.08)	0.04	−1.55	(−2.90; −0.23)	0.02	−1.55	(−2.87; −0.24)	0.02
SF-36 PCS^b^	1.34	(−0.76; 3.45)	0.21	1.36	(−0.72; 3.48)	0.20	1.27	(−0.81; 3.40)	0.23
SF-36 MCS^b^	1.65	(−1.53; 4.85)	0.31	2.06	(−1.07; 5.27)	0.19	2.30	(−0.64; 5.25)	0.12
FAB^a^	−11.84	(−20.58; −3.18)	0.008	−11.71	(−20.29; −3.17)	0.008	−11.55	(−20.10; −2.95)	0.009

Average treatment effect on the treatment targets and secondary outcome measures based on an unadjusted and adjusted linear mixed model.

Abbreviations: B-IPQ = Brief Illness Perception Questionnaire; BRIQ-lim = Behavioural Response to Illness Quetionnarie, subscale “limiting behaviour”; BRIQ-all = Behavioural Response to Illness Quetionnarie, subscale “all or nothing behaviour”; SCL-8 = Symptom Checklist-8; SF-36 = Short Form 36 Health Survey (PCS = physical component summary, MCS = mental component summary); FAB = Fear Avoidance Behaviour.

Higher scores on the following questionnaires indicate less symptoms/better function: SF-36 PCS and MCS; Higher scores on the following questionnaires indicate more symptoms/worse function: B-IPQ, BRIQ, SCL-8, FAB and Whiteley 6.

a The sum of all items was transformed into a 0–100 scale to enable comparison between scales.

b The sum scores of SF-36 were converted into T-scores based on US normative data.

c Basic adjustment: Adjusted for age, sex, recruitment (ED/referred from GP), and previous mental health status (i.e. previous psychiatric diagnosis, yes/no).

d Full adjustment: Adjusted for basic adjustments and education level, functional somatic syndrome diagnosis prior to injury (yes/no), current psychological distress (SCL-8).

The difference in change between treatment groups favoured GAIN + EUC across all treatment targets (Table 3). At 3-month follow-up the average difference in change on B-IPQ (illness perceptions) was −10.3 (95% CI −14.8 to −5.4, *p* < 0.001); on BRIQ-limiting behaviour −5.9 (95% CI −11.5 to −0.3, *p* = 0.04); on BRIQ-all or nothing behaviour −7.8 (95% CI −12.8 to −2.7, *p* = 0.003); and on FAB (fear avoidance behaviour) −11.6 (95% CI −20.1 to −3.0, *p* = 0.009).

Among the secondary outcomes, change in illness worry favoured GAIN + EUC with a difference in change of −1.6 on Whiteley-6 (95% CI −2.9 to −0.2, *p* = 0.02), whereas differences in physical and mental health were smaller and their confidence intervals included the possibility of no difference (Table 3).

### Additional measures

Participants expressed high satisfaction with the GAIN intervention, the therapists delivering the intervention, and the collaboration among professionals on the ESQ (Figure S2, Supplemental Materials). Most participants (n = 137, 69%) reported a subjective improvement in their general health following the intervention (Figure S3, Supplemental Materials).

A total of 7/148 participants (5%) who provided data at end of intervention in the GAIN + EUC group reported minor adverse effects of the intervention such as temporary fatigue or other symptom increase due to participating in intervention sessions, filling out questionnaires or gradually increasing hours at work. No serious adverse effects were reported.

## Discussion

In this stepped wedge randomised trial we evaluated the effectiveness of GAIN, an interdisciplinary, cognitive behavioural intervention, added to EUC in reducing PPCS in adults (18–60 years) when delivered by therapists in a municipality setting. At 3 months after intervention, the difference in change in PPCS between treatment groups favoured the group receiving GAIN added to EUC, but the treatment effect failed to reach the 0.05 threshold for statistical significance. Notably, the GAIN + EUC group reported a substantially greater reduction in symptoms than the EUC group at end of intervention, but the difference between groups diminished over time. Moreover, GAIN + EUC had substantially larger reductions at 3-months follow-up than the EUC group in maladaptive, symptom-related perceptions and behaviours (i.e. the predefined intervention targets) and in illness worry (one of the secondary outcomes). There was no difference in treatment effect on psychological distress or physical and mental health. The participants reported high satisfaction with GAIN, which was also reflected by their high adherence to participating in the treatment sessions, suggesting that the intervention was helpful and meaningful for them. In addition, we demonstrated that it was feasible to implement GAIN in a municipality setting, and to educate therapists to deliver the intervention as intended.

Of note, most participants were women, and a high percentage had a higher education. This is consistent with previous clinical PPCS cohorts and with evidence indicating that women and people with higher education are at increased risk of developing PPCS.^26^

The findings regarding the non-significant treatment effect of GAIN on PPCS in the present trial are in contrast to findings from the GAIN 1.0 trial.^11^ One explanation may be the tendency for a treatment program's efficacy to diminish when it is scaled up, and when an intervention is delivered under the usual conditions in which it will be applied, and not under more ideal conditions, which is sometimes referred to as “the voltage drop”. In the present trial, the treatment effect of GAIN may have been reduced compared to the GAIN 1.0 trial due to a series of factors. GAIN is a complex intervention including a “toolbox” with several different intervention strategies targeting different symptom-perpetuating factors, which the therapists may apply flexibly and adapt to the individual patient without a fixed treatment-algorithm. This place demands on the therapists' experience and skills, and it may require more training to deliver the GAIN intervention with the same quality as the more specialised therapists in the GAIN 1.0 trial. In GAIN 2.0, the therapists only had a brief two-day course in concussion, PPCS and the GAIN intervention, and no time to practice and to develop their competencies before delivering GAIN to participants in the clinical trial. This may explain the low therapist adherence observed in the audio recordings for strategies targeting maladaptive, symptom-perpetuating perceptions–a key component of GAIN–which may be particularly challenging for therapists without prior experience in third-wave cognitive behavioural therapy. As explained in the introduction, maladaptive perceptions are associated with maladaptive illness behaviour and PPCS. Therefore, it may be speculated that the effect of GAIN could be improved by more therapist training in intervention strategies targeting symptom-perpetuating illness perceptions (Table S3, Supplemental Materials). Another important factor may be that the participants in GAIN 2.0 were more complex and predisposed for a poorer outcome than the participants in GAIN 1.0. Age above 30 is associated with worse outcome after concussion compared to adolescents and young adults.^2^^,^^27^ Moreover, a higher number of participants in GAIN 2.0 compared to GAIN 1.0 had at some timepoint in life prior to the concussion been diagnosed with either a psychiatric disorder or a functional somatic syndrome disorder, which are known negative predictors for outcome.^27^, ^28^, ^29^ In line with this, we found that the average treatment effect in GAIN 2.0 varied significantly across participants with and without a psychiatric diagnosis prior to the concussion. The combination of less experienced therapists and more complex participants would be expected to result in a smaller effect of the intervention.

It may also be that an 8-week, low dose intervention is not sufficient for a significant number of highly symptomatic patients with PPCS. The mean symptom-level at end of intervention was still above our suggested cut-off for treatment demanding symptoms (RPQ ≥20). Thus, the intervention may have ended at a time when many participants were in recovery, yet vulnerable to symptom worsening, and could have benefitted from more treatment sessions. Some participants might also have benefited from receiving more comprehensive psychotherapy from mental health specialists. This hypothesis is supported by prior studies demonstrating that cognitive behavioural therapy delivered by psychologists may be effective in reducing PPCS.^13^

Descriptively, there was a symptom reduction in both treatment groups from baseline to end of intervention. This may reflect spontaneous recovery, but since patients with PPCS at 2 months are at risk of still having PPCS after a year, it may be speculated that the improvement in the EUC group is partly owing to the combined effect of the clinical assessment and EUC.^30^ An accurate diagnosis, and provision of evidence-based information, reassurance, and advice about PPCS may in itself improve recovery.^4^ EUC in the present study was more comprehensive than usual care of patients with PPCS, which may have attenuated the estimated effect of GAIN. However, there are mixed findings regarding the stand-alone effects of this type of intervention as compared to more comprehensive psychological and rehabilitative approaches.^31^ Notably, participants in the GAIN + EUC group had faster symptom improvement than the EUC group, which might be owing to the GAIN intervention speeding up the recovery process. However, our study design in both GAIN studies makes it impossible to know how much of the GAIN treatment effect is attributable to expectancy and non-specific attention. Thus, placebo effects may have contributed to improved symptoms in both the GAIN + EUC and the EUC group.

In both GAIN studies, there was a substantial reduction in maladaptive, symptom-related perceptions and behaviours, i.e. the predefined treatment targets. This is an important finding, since maladaptive perceptions and behaviours are associated with worse outcomes after concussion, and interventions modifying these may hold the potential to improve recovery.^7^^,^^8^

GAIN includes treatment principles from third-wave cognitive behavioural therapy that encourage the patient to accept a tolerable degree of physical symptoms or unpleasant thoughts and feelings (fx. catastrophising and anxiety) to be able to engage in meaningful everyday activities. In GAIN, symptom reduction is considered a likely long-term byproduct of challenging oneself at an appropriate level and thereby increasing the tolerance threshold. Therefore, it may be discussed if symptom reduction as measured by the RPQ is the most relevant primary outcome for measuring the effect of GAIN, compared to for example a measure of participation in everyday life activities or qualitative data, as the RPQ may not capture the effect of the intervention on recovery. In line with this, a feasibility study of a third-wave cognitive behavioural intervention for PPCS applied qualitative interviews to evaluate the intervention and found that the intervention had a positive impact on recovery.^32^

Our study has some limitations. The inclusion of only five clusters (i.e. only 5 units of randomisation) in a stepped-wedge randomised trial limits statistical power regardless of a high number of participants making it difficult to detect a possible significant effect of the intervention. Therapists from the latest randomised clusters gained limited experience with GAIN, and although this was addressed in the analysis, it may have influenced patient outcomes. There was a low response rate at primary outcome which may have introduced bias. Many trials are analysed using the strong and unlikely assumption that data are missing completely at random (MCAR), such as complete-case analyses. The repeated measures linear mixed model we used only assume that data are missing at random (MAR), meaning that the missing data mechanism is related to observed data only and not unobserved data. Under these circumstances the methods we implemented provide unbiased estimates.^24^ Like any other RCT study of human subjects having missing information, the missing at random assumption cannot be verified empirically. Some studies have shown that data missing not at random (MNAR) are modelled well using linear mixed models.^24^ Ideally, more effort should be put into ensuring fewer missing data than the current study, where the substantial loss of information reduces power and robustness of the estimated treatment effects. Due to the nature of the intervention, masking of participants and therapists regarding treatment allocation was not possible. Moreover, the study was conducted in a Danish municipality setting and this should be considered when assessing the generalisability of the findings to populations with other linguistic, cultural, or ethnic backgrounds. Finally, potential adverse effects of the intervention could have been monitored more proactively.

Our trial also has several strengths. To the authors' knowledge, this is the largest randomised controlled trial that has been conducted to date assessing an interdisciplinary intervention for patients with PPCS. We recruited 310 participants across a broad age range, both diagnosed at the hospital and referred from GPs, and we used broad inclusion criteria (i.e. not excluding patients with mild to moderate co-morbidity), thereby increasing the generalisability of the results. All potential participants went through a thorough clinical assessment by a physician verifying the concussion diagnosis using standardised criteria. The intervention was delivered close to the participants’ home with the possibility of video consultations, ensuring that GAIN was easily accessible and interfered less with daily life. The content of the intervention and treatment dose was flexible and tailored to individual needs. Furthermore, the pragmatic study design demonstrated that it was feasible for therapists in the municipalities to deliver GAIN as intended, and to implement the GAIN intervention in the healthcare system through a collaboration between municipalities.

In conclusion, data from the present study could not confirm the hypothesis that GAIN added to EUC led to larger long-term reduction in PPCS than EUC alone in adults. However, GAIN may speed up the recovery process and may improve long-term prognosis by reducing symptom-related perceptions and behaviours. Moreover, GAIN was feasible in the municipalities, and patient satisfaction was high.

Future studies should explore if GAIN increase participation in everyday life despite PPCS and thereby improve long-term recovery. Also, future studies should explore whether enhanced therapist training and the possibility to extend the intervention period or to provide booster sessions in a follow-up period for the most vulnerable participants may improve the long-term effect of GAIN. More studies in effective interventions for PPCS are needed that may inform an improvement of the GAIN intervention. As more evidence for effective treatment emerges, it will be possible to improve the intervention by expanding the GAIN toolbox.

## Contributors

Authors’ contributions according to the CRediT taxonomy and in alphabetical order:

Conceptualisation: CUR, HMR, HHS, JFN, LO, MMT, NDS, TS.

Data curation: CBP, CN, LAH, LO, PPE.

Formal analysis: CBP, CN, LAH, LO.

Funding acquisition: HHS, JFN, LO, MMT.

Investigation: CBP, HHS, JFN, LAH, LO, MMT, PPE.

Methodology: CUR, HMR, HHS, JFN, LAH, LO, MMT, NDS, PPE, TS.

Project administration: HHS, LAH, LO, MMT, JFN.

Writing–original draft: LAH, MMT.

Writing—review and editing: CUR, CBP, CN, HHS, HMR, JFN, LAH, LO, MMT, NDS, PPE, TS.

All authors have read and approved the final version of the manuscript.

The first authors (LAH + MMT) and the last author (JFN) had full access to and verified all the data in the study and had final responsibility for the decision to submit for publication. No author has been paid to write this article by any agency.

## Data sharing statement

Individual, de-identified participant data that underlie the results reported in this article will be available beginning 9 months and ending 36 months following article publication. Data will be shared for individual participant data meta-analysis with investigators whose proposed use of data has been approved by an independent review committee identified for this purpose. The study protocol is available at https://www.fagperson.hospitalsenhedmidt.dk/afdelinger/regionshospitalet-hammel-neurocenter/university-research-clinic/forskning-om-hjernerystelse/gain/. Proposals for accessing data may be send by e-mail to professor, DMSc, Joergen Feldbaek Nielsen, Hammel Neurorehabilitation and Research Centre: joerniel@rm.dk. Data will be available on the RED-Cap platform hosted at Aarhus University, Denmark.

According to Danish and EU data protection legislation, sensitive individual-level health data may not be posted in an unrestricted, open-science repository. This data sharing statement fulfils the requirements from The International Committee of Medical Journal Editors (ICMJE).

## Declaration of interests

LAH, MMT, HHS and LO received grants from Sygeforsikringen “danmark” during the conduct of the study. Payment was made to Hammel Neurorehabilitation and Research Centre. Central Denmark Region Provided financial support to the outpatient clinic at Hammel Neurorehabilitation Centre. All other authors received no support for the present manuscript.

In the past 36 months, CUR has received grants from the Independent Research Fund Denmark, Aage and Johanne Louis-Hansen Foundation, Tryg Foundation, The Psychiatric Research Foundation, Central Denmark Region, Lundbeck Foundation and Central Denmark Region Research Fund, and royalties from the publisher Gyldendal A/S. In addition, CUR is an Advisory Board Member for the German trial: iSOMA+, and a board member of European Association for Psychosomatic Medicine (EAPM). CUR has received payment for expert testimony from the Einstein Foundation Berlin, the Independent Research Fund Denmark (DFF), the Trondheim Mohn Research Foundation, the Icelandic Research Fund, the Australian National Health and Medical Research Council (NHMRC), and the Irish Research Council.

In the past 36 months, NDS declare that his institution has received research grants or contracts from CIHR, Mitacs, Canada Foundation for Innovation, WorkSafeBC and VGH + UBC hospital foundation. NDS declares that a collaborator's institution has received research grants or contracts from Ontario Brain Institute and DoD. NDS (Dr. Noah Silverberg Inc) has received consulting fees for clinical and medical-legal consulting. Furthermore, NDS has received honorarium for lectures at Canadian Society of Medical Evaluators, WorkSafeBC, Yukon Workers' Compensation Health and Safety Board, Worker's Compensation Board of Alberta, NYU-Langone, International Academy of Independent Medical Examiners, and University of Calgary.

CBP, CN, HMR, PPE, and TS declare no relationships/activities/interests related to the content of this manuscript.

## Appendix A Supplementary data

Supplementary Figures and Tables

Study Protocol

## References

[1] Cancelliere C., Verville L., Stubbs J.L. (2023). Post-concussion symptoms and disability in adults with mild traumatic brain injury: a systematic review and meta-analysis. J Neurotrauma.

[2] Graff H.J., Siersma V., Møller A. (2019). Premorbid risk factors influencing labour market attachment after mild traumatic brain injury: a national register study with long-term follow-up. BMJ Open.

[3] Galili S.F., Bech B.H., Vestergaard C. (2017). Use of general practice before and after mild traumatic brain injury: a nationwide population-based cohort study in Denmark. BMJ Open.

[4] Rytter H.M., Graff H.J., Henriksen H.K. (2021). Nonpharmacological treatment of persistent postconcussion symptoms in adults: a systematic review and meta-analysis and guideline recommendation. JAMA Netw Open.

[5] Hou R., Moss-Morris R., Peveler R., Mogg K., Bradley B.P., Belli A. (2012). When a minor head injury results in enduring symptoms: a prospective investigation of risk factors for postconcussional syndrome after mild traumatic brain injury. J Neurol Neurosurg Psychiatry.

[6] Clark C.N., Edwards M.J., Ong B.E. (2022). Reframing postconcussional syndrome as an interface disorder of neurology. Brain.

[7] Silverberg N.D., Mikolić A. (2023). Management of psychological complications following mild traumatic brain injury. Curr Neurol Neurosci Rep.

[8] Mah K., Hickling A., Reed N. (2018). Perceptions of mild traumatic brain injury in adults: a scoping review. Disabil Rehabil.

[9] Thastum M.M., Rask C.U., Naess-Schmidt E.T. (2018). Design of an early intervention for persistent post-concussion symptoms in adolescents and young adults: a feasibility study. NeuroRehabilitation.

[10] Henningsen P., Gundel H., Kop W.J., EURONET-SOMA Group (2018). Persistent physical symptoms as perceptual dysregulation: a neuropsychobehavioral model and its clinical implications. Psychosom Med.

[11] Thastum M.M., Rask C.U., Næss-Schmidt E.T. (2019). Novel interdisciplinary intervention, GAIN, vs. enhanced usual care to reduce high levels of post-concussion symptoms in adolescents and young adults 2–6 months post-injury: a randomised trial. eClinicalMedicine.

[12] Lal A., Kolakowsky-Hayner S.A., Ghajar J., Balamane M. (2018). The effect of physical exercise after a concussion: a systematic review and meta-analysis. Am J Sports Med.

[13] Möller M., Lexell J., Wilbe Ramsay K. (2021). Effectiveness of specialized rehabilitation after mild traumatic brain injury: a systematic review and meta-analysis. J Rehabil Med.

[14] Naess-Schmidt E.T., Thastum M.M., Stabel H.H. (2022). Interdisciplinary intervention (GAIN) for adults with post-concussion symptoms: a study protocol for a stepped-wedge cluster randomised trial. Trials.

[15] Carroll L.J., Cassidy J.D., Holm L., Kraus J., Coronado V.G., WHO Collaborating Centre Task Force on Mild Traumatic Brain Injury (2004). Methodological issues and research recommendations for mild traumatic brain injury: the WHO Collaborating Centre Task Force on Mild Traumatic Brain Injury. J Rehabil Med.

[16] Zeldovich M., Bockhop F., Covic A. (2022). Reference values for the rivermead post-concussion symptoms questionnaire (RPQ) from general population samples in the United Kingdom, Italy, and the Netherlands. J Clin Med.

[17] Ruff R.M., Iverson G.L., Barth J.T. (2009). Recommendations for diagnosing a mild traumatic brain injury: a National Academy of Neuropsychology education paper. Arch Clin Neuropsychol.

[18] King N.S., Crawford S., Wenden F.J., Moss N.E., Wade D.T. (1995). The Rivermead Post Concussion Symptoms Questionnaire: a measure of symptoms commonly experienced after head injury and its reliability. J Neurol.

[19] Broadbent E., Petrie K.J., Main J., Weinman J. (2006). The brief illness perception questionnaire. J Psychosom Res.

[20] Spence M., Moss-Morris R., Chalder T. (2005). The Behavioural Responses to Illness Questionnaire (BRIQ): a new predictive measure of medically unexplained symptoms following acute infection. Psychol Med.

[21] Carstensen T.B.W., Ørnbøl E., Fink P. (2020). Detection of illness worry in the general population: a specific item on illness rumination improves the Whiteley Index. J Psychosom Res.

[22] Christensen K.S., Fink P., Toft T., Frostholm L., Ørnbøl E., Olesen F. (2005). A brief case-finding questionnaire for common mental disorders: the CMDQ. Fam Pract.

[23] Hemming K., Taljaard M., Forbes A. (2017). Analysis of cluster randomised stepped wedge trials with repeated cross-sectional samples. Trials.

[24] Bell M.L., Rabe B.A. (2020). The mixed model for repeated measures for cluster randomized trials: a simulation study investigating bias and type I error with missing continuous data. Trials.

[25] Li F., Hughes J.P., Hemming K., Taljaard M., Melnick E.R., Heagerty P.J. (2021). Mixed-effects models for the design and analysis of stepped wedge cluster randomized trials: an overview. Stat Methods Med Res.

[26] Madsen B.Å., Fure S.C.R., Røe C. (2025). The relationship between personality traits and health-related quality of life after mild-to-moderate traumatic brain injury. BMC Neurol.

[27] van der Naalt J., Timmerman M.E., de Koning M.E. (2017). Early predictors of outcome after mild traumatic brain injury (UPFRONT): an observational cohort study. Lancet Neurol.

[28] Cassidy J.D., Cancelliere C., Carroll L.J. (2014). Systematic review of self-reported prognosis in adults after mild traumatic brain injury: results of the international collaboration on mild traumatic brain injury prognosis. Arch Phys Med Rehabil.

[29] Green K., Cairncross M., Panenka W.J., Stubbs J.L., Silverberg N.D. (2021). History of functional somatic syndromes and persistent symptoms after mild traumatic brain injury. J Neuropsychiatry Clin Neurosci.

[30] Theadom A., Parag V., Dowell T., BIONIC Research Group (2016). Persistent problems 1 year after mild traumatic brain injury: a longitudinal population study in New Zealand. Br J Gen Pract.

[31] Heslot C., Azouvi P., Perdrieau V., Granger A., Lefèvre-Dognin C., Cogné M. (2022). A systematic review of treatments of post-concussion symptoms. J Clin Med.

[32] Faulkner J.W., Chua J., Voice-Powell A. (2025). Experience of Acceptance and Commitment Therapy for those with mild traumatic brain injury (ACTion mTBI): a qualitative descriptive study. PLoS One.

