# Supplementary material for: Interdisciplinary intervention (GAIN) vs. enhanced usual care for reducing severe post-concussion symptoms in adults 2–4 months post-injury: a stepped wedge cluster randomised controlled trial
Source: eClinicalMedicine. 2026 Sep 22;100:104243. doi: 10.1016/j.eclinm.2026.104243 (PMC13672966; doi:10.1016/j.eclinm.2026.104243)
Supplement: Supplementary file 1 [file mmc1.pdf]

Supplemental Materials

Interdisciplinary intervention (GAIN) vs. enhanced usual care for reducing severe post-concussion symptoms in adults 2–4 months post-injury: A stepped wedge cluster randomised controlled trial

Line Amalie Hellemose, MD [a*]; Mille Møller Thastum, PhD [a*†]; Henriette Holm Stabel, PhD [a]; Lene Odgaard, PhD [a]; Charlotte Ulrikka Rask [b,c]; Noah D. Silverberg, PhD [d,e,f]; Toril Skandsen, PhD [g,h]; Hana Malá Rytter, PhD [i,j,k]; Charlotte Nygaard MD [a]; Peter Preben Eggertsen, PhD [l]; Carsten B. Pedersen, DMSc [a,m]; Jørgen Feldbæk Nielsen, DMSc [a].

[a] Hammel Neurorehabilitation Centre and University Research Clinic, Aarhus University, Aarhus, Denmark.

[b] Department of Child and Adolescent Psychiatry, Aarhus University Hospital, Aarhus N, Denmark

[c] Department of Clinical Medicine, Aarhus University, Aarhus N, Denmark

[d] Department of Psychology, University of British Columbia, Vancouver, BC, Canada

[e] Rehabilitation Research Program, Centre for Aging SMART, Vancouver Coastal Health Research Institute, Vancouver, BC, Canada

[f] Member, Djavad Mowafaghian Centre for Brain Health, Vancouver, BC, Canada

[g] Department of Neuromedicine and Movement Science, Norwegian University of Science and Technology (NTNU), Trondheim, Norway.

[h] Clinic of Rehabilitation, St. Olav's Hospital, Trondheim University Hospital, Trondheim, Norway.

[i] The Danish Concussion Center, Copenhagen, Denmark

[j] Department of Neurology, University Hospital Bispebjerg - Frederiksberg, Copenhagen, Denmark

[k] Department of Psychology, University of Copenhagen, Copenhagen, Denmark

[l] Department of Infectious Diseases, Aalborg University Hospital, Aalborg, Denmark

[m] National Centre for Register-Based Research, Aarhus University, Aarhus V, Denmark

* Joint first authors.

† Corresponding author. E-mail address: Mille.Moeller.Thastum@auh.rm.dk (M.M. Thastum).

List of contents:

Figure S1: Diagram of the stepped-wedge cluster randomised design.

Figure S2. Results from the Experience of Service Questionnaire.

Figure S3. Results from the Clinical Global Impression Scale.

Figure S4. Scatterplots and histograms of Rivermead Post-concussion symptoms Questionnaire (total score).

Table S1. Overview of the structure and content of GAIN**.**

Table S2. Overview of outcome measures and measurement points.

Table S3. Therapists' adherence to the GAIN treatment manual.

Table S4. Difference in change between GAIN+EUC and EUC in RPQ total score: per-protocol analysis.

Table S5: Difference in change between GAIN+EUC and EUC in RPQ total score by predefined potential confounders.

	**2021**		**2021/2022**	**2022**			**2022/2023**
**Recruitment period**	**1 May – 31 July**	**1 August – 31 October**	**1 November - 31 January**	**1 February – 30 April**	**1 May – 31 July**	**1 August – 31 October**	
**Intervention period**		**1 August – 31 October**	**1 November - 31 January**	**1 February – 30 April**	**1 May – 31 July**	**1 August – 31 October**	**1 November - 31 January**
**1. Randomisation**		Comparison	Cluster 1	Cluster 1	Cluster 1	Cluster 1	Cluster 1
**2. Randomisation**		Comparison	Comparison	Cluster 2	Cluster 2	Cluster 2	Cluster 2
**3. Randomisation**		Comparison	Comparison	Comparison	Cluster 3	Cluster 3	Cluster 3
**4. Randomisation**		Comparison	Comparison	Comparison	Comparison	Cluster 4	Cluster 4
		Comparison	Comparison	Comparison	Comparison	Comparison	Cluster 5

Figure S1: Diagram of the stepped-wedge cluster randomised design. There were five clusters (i.e. the health-clusters of the municipalities in Central Denmark Region) and six recruitment periods of each 3 months length. Patients from all clusters were included in all recruitment periods and were allocated to GAIN+EUC or EUC depending on the patient's place of residence (i.e. which health-cluster). The white boxes illustrate clusters in the comparison condition only (EUC), and the blue boxes illustrate clusters providing the intervention in addition to EUC (GAIN + EUC). Recruitment of patients to the comparison condition started 3 months before beginning clinical assessments (1 May – 31 July 2021). Recruitment, clinical assessment, EUC intervention to patients in the comparison condition, and education of therapists from the first health-cluster in the GAIN intervention took place in a 3 month period before the GAIN-intervention started in the first cluster (1 August – 31 October 2021).

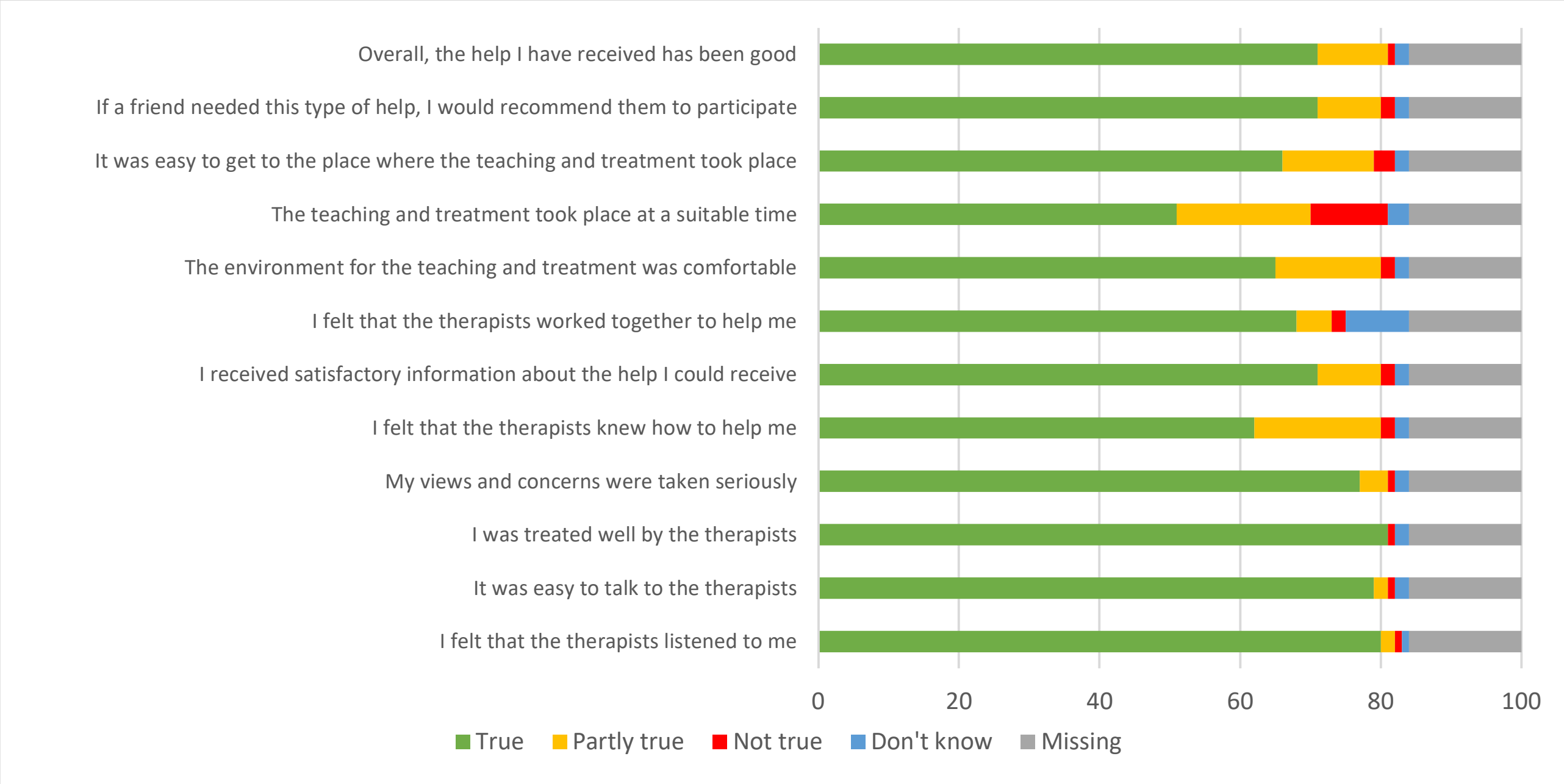

Figure S2. Results from the Experience of Service Questionnaire. Applied to patients allocated to GAIN+EUC (n=170) at end of intervention (missings: n=27, 16%).

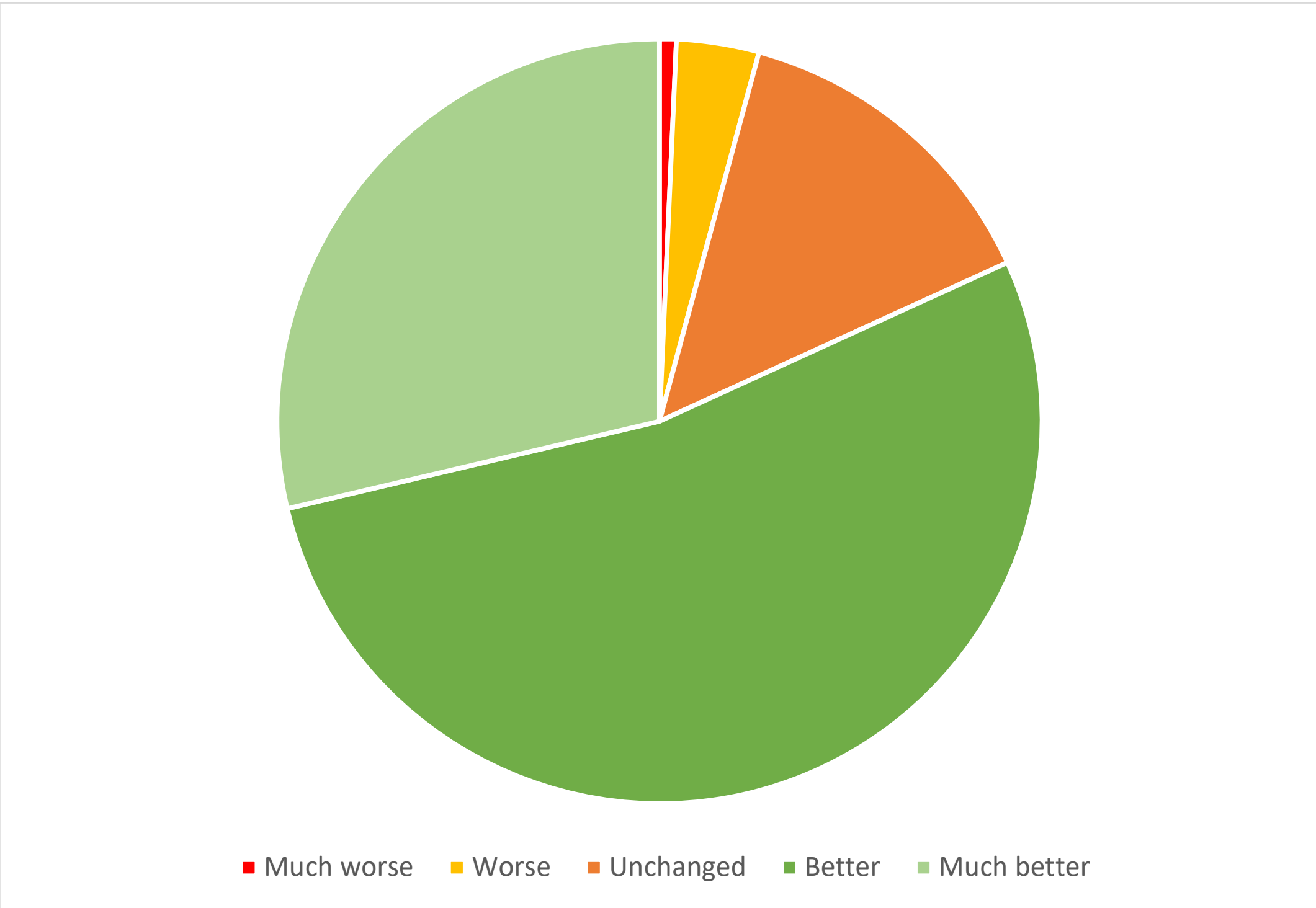

Figure S3. Results from the Clinical Global Impression Scale. Subjective improvements in the state of health measured in the GAIN+EUC group (n=143/170, 27 missings). Patients were asked: “Compared to before you started treatment, how would you describe your health today?”

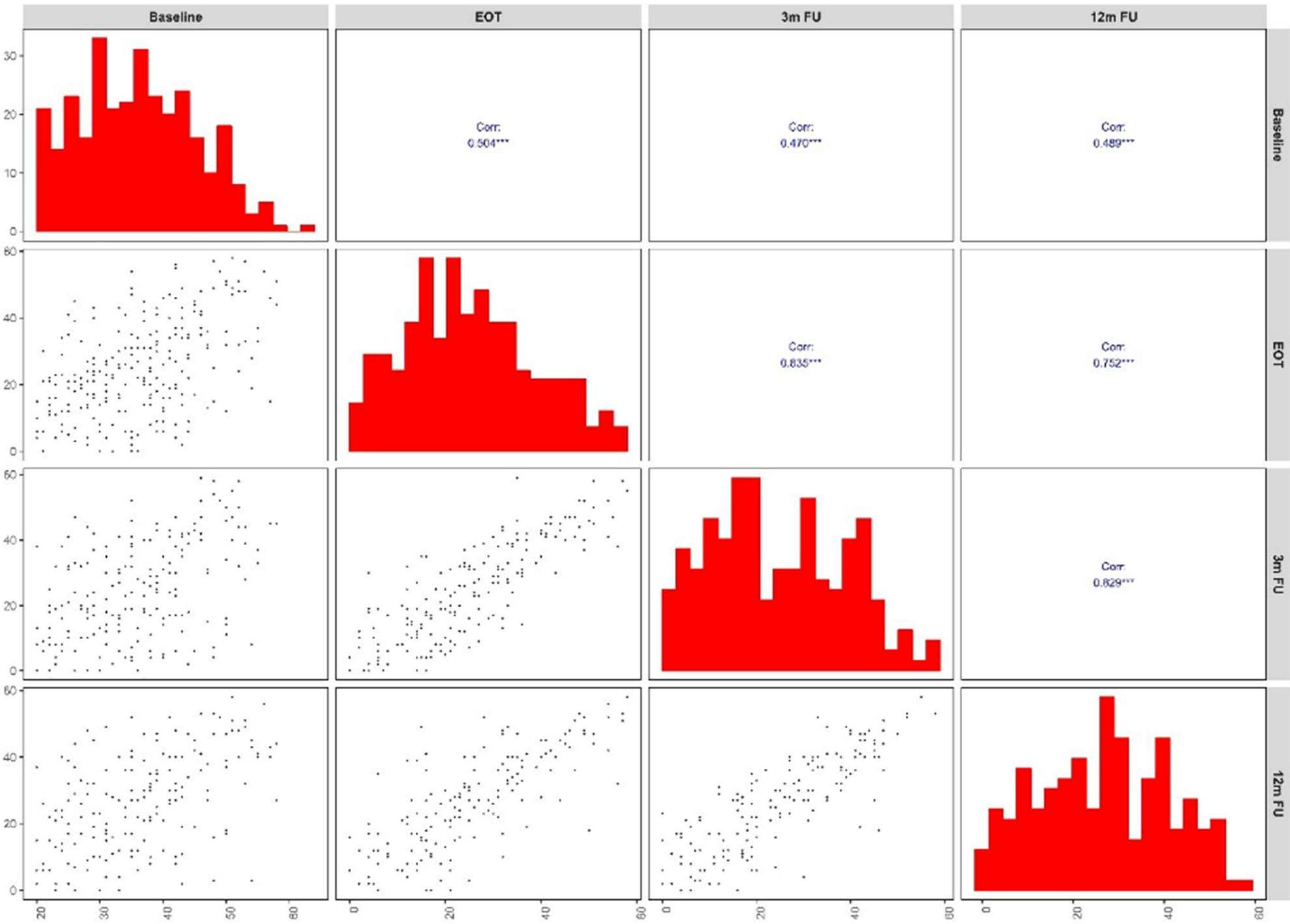

Figure S4. Scatterplots and histograms of Rivermead Post-concussion symptoms Questionnaire (total score) for all participants (GAIN+EUC and EUC) at "baseline", "end of intervention", "3-months follow up" and "12-months follow-up" along with pairwise Pearson correlation coefficient. The histograms indicate that data may be assumed to be normally distributed. As expected, RPQ measures are correlated within subjects, and correlation is highest for measurements closest in time.

**Session 1**<br>**Group**<br>**(2 hours)**<br><br>Neuropsychologist<br>Occuptional therapist<br>Physiotherapist<br><br>(Relatives invited)	**Session 2**<br>**Group (1 hour)**<br>**Individual (30 min)**<br><br>Neuropsychologist<br>Occupational therapist<br>Physiotherapist<br><br>(Relatives invited)	**Session 3-7**<br>**Individual**<br>**(approx. 30 min)**<br><br>Occupational therapist or<br>Physiotherapist (individually allocated)<br><br>In person or by videoconference	**Session 8**<br>**Group**<br>**(2 hours)**<br><br>Neuropsychologist<br>Occupational therapist<br>Physiotherapist
**Psychoeducation, topics:**<br><br>Introduction to GAIN<br><br>What is a concussion?<br><br>The bio-psycho-social understanding of PPCS<br><br>The importance of physical activity<br><br>**Group-based physical exercise**<br>Stair climbing	**Psychoeducation, topics:**<br><br>Chronic stress after concussion and its consequences<br><br>The autonomic nervous system.<br><br>**Group-based exercise**<br>Diaphragmatic breathing		**Evaluating the GAIN intervention**<br><br>**Mutual exchange of experience and advice**<br><br>**”Where do I want to go from here?**<br>Future goals and next steps
	**First individual consultation:**<br><br>Establish baseline physical activity<br><br>Formulating goals, building a “step ladder”	**Consultations with primary therapist**<br><br>Physical attendance or video consultation	
**Homework**<br>1. Baseline physical activity: evaluate intensity by using the BORG scale.<br><br>2. Questionnaire: personally meaningful activities	**Homework**<br>Individually tailored training program based on the GAIN manual working towards the personally meaningful goals	**Homework**<br>Individually tailored training program based on the GAIN manual working towards the personally meaningful goals	

Table S1. Overview of the structure and content of GAIN.
Each group had a maximum of 9 participants. The group sessions were mandatory, but the number of individual sessions was flexible (a maximum of six), and adjusted according to the patient’s needs and wishes.

Table S2. Overview of outcome measures and measurement points.

Measurement points (approx. months after concussion)		T0: Baseline (4 months)	T1: End of intervention (8 months)	T2: 3 months follow-up (11 months, (primary endpoint)	T3: 12 months follow-up (20 months)
Primary outcome	RPQ	x	x	x	x
Treatment targets	B-IPQ	x	x	x	
	BRIQ*	x	x	x	
	FAB	x	x	x	
Secondary outcomes	SF-36**	x		x	
	Whiteley-6	x		x	
	SCL-8	x		x	
Additional measures	CGI		x (GAIN+EUC only)	x (GAIN+EUC only)	
	ESQ		x (GAIN+EUC only)		
	Adverse events		x		
	Other non-farmacological treatment			x	

Abbreviations: RPQ = Rivermead Post-concussion Questionnaire; B-IPQ = Brief Illness perception Questionnaire; BRIQ = Behavioral Response to Illness Questionnaire; ESQ=Experience of Service Questionnaire; FAB = Fear Avoidance Behavior measure; SF-36 = The Short Form 36 Health Survey; SCL-8 = Symptom Checklist-8; CGI = The Clinical Global Impression Scale

* Two subscales: “Limiting behavior” and “all-or-nothing” behavior

** Physical and Mental Component Summaries

Table S3. Therapists' adherence to the GAIN treatment manual

<table>
<tr><th>Individual session number
(sessions evaluated)</th><th>Intervention target</th><th>Intervention method / therapist action</th><th>Frequency
(% of sessions)</th></tr>
<tr><td rowspan="2">1st individual session
(10%=14 sessions)</td><td>Establishing baseline physical activity level</td><td>Evaluating the patients baseline level of physical activity in everyday life (duration and intensity of activity according to the Borg Rating of Perceived Exertion (the BORG scale)</td><td>100 %</td></tr>
<tr><td>Goal formulation based on meaningful everyday activities</td><td>Identify personally meaningful goals based on a questionnaire about challenges in everyday activities (homework between 1. and 2. group session). Explain how to use a "step ladder" to achieve the goals (i.e. graduated return to activities)</td><td>100 %</td></tr>
<tr><td rowspan="4">2nd – 7th individual session
(10%=65 sessions)</td><td>Physical activity</td><td>Evaluating / adjusting physical activity in everyday life</td><td>90 %</td></tr>
<tr><td>Goal formulation based on meaningful everyday activities</td><td>Evaluating / adjusting goals, identifying the next step on the step ladder.</td><td>86 %</td></tr>
<tr><td>Maladaptive symptom-perpetuating perceptions</td><td>Providing education on
• concussion and transient symptoms as part of the normal recovery process.
• persistent PPCS in terms of the bio-psycho-social disease model.

Using techniques derived from Acceptance and Commitment Therapy such as
• creating a distance to one's thoughts.
• exploring the relationship between thoughts and behaviour.
• identifying individually important goals.
• refocusing on personally meaningful goals rather than focusing on symptoms.</td><td>57 %</td></tr>
<tr><td>Maladaptive symptom perpetuating behaviour</td><td>Providing education on:
• the negative effects of excessive rest, physical or mental exertion, and "all-or-nothing" behaviour.
• the negative effects of fear-avoidance behaviour.
• the negative effects of "pushing through symptoms" and how it may exacerbate symptoms.
• the health-related benefits of exercise.
• the risk of chronic stress after concussion and its consequences (lower threshold for autonomic stress reactions, the self-perpetuating circle of stress).

Supporting the patient in balancing activity and rest at home, school, and / or work by:
• increasing or reducing daily[1] activities.
• managing and prioritizing energy and resources, also including pleasurable activities.
• scheduling daily activities[1] and evening out the daily / weekly activity level

Supporting the patient in gradually returning to activities by:
• planning how to gradually increase daily activities[1] and physical exercise in duration and intensity.
• using a "pyramid of objectives" to work stepwise towards personally meaningful goals.</td><td>87 %</td></tr>
</table>

		Supporting the patient in relaxation training by using<br>• diaphragmatic breathing exercise.<br>• body scan meditation apps.	

[1] Daily activities are broadly defined as everything we normally do in daily living including both physical and mental activities in work and education, leisure time, and personal care.

The table provides an overview of the intervention targets and the possible methods and therapist actions provided in the GAIN manual. In the individual sessions, the therapists may choose from the different methods, adapting the intervention to the individual patient's symptom perpetuating perceptions and behaviour, and the patient's goal formulation.

All individual sessions were audio recorded. An occupational therapist, who completed the two-day training course in the GAIN manual, but was not involved in the delivery of GAIN, listened to a random sample of 10% of the sessions (1st session: n=14; 2nd -7th session: n=65) in order to check if the therapists adhered to the treatment manual and applied the methods from GAIN. The results are presented in the far-right column.

The first session was different from the following sessions. The goal was primarily to get information on the patient's baseline physical activity and identify personally meaningful goals for the individual patient based on a questionnaire filled out by the patient (homework between the two first group sessions).

Table S4. Difference in change between GAIN+EUC and EUC in RPQ total score from baseline to end of treatment, 3-month follow-up, and 12-month follow-up in the per-protocol analysis (n=279).

	**Unadjusted**			**Basic adjustment***			**Full adjustment****		
**Outcome**	**Mean**	**95 % CI**	**p**	**Mean**	**95 % CI**	**p**	**Mean**	**95 % CI**	**p**
RPQ, total (0-64)									
End of treatment	-5.43	(-8.20; -2.75)	<0.001	-5.54	(-8.37; -2.91)	<0.001	-5.66	(-8.48; -3.09)	<0.001
3 months follow-up	-2.60	(-5.55; 0.24)	0.07	-2.74	(-5.76; 0.04)	0.05	-2.72	(-5.71; 0.01)	0.05
12 months follow-up	-1.85	(-4.99; 1.24)	0.24	-1.90	(-5.11; 1.12)	0.20	-2.00	(-5.19; 0.96)	0.18

Average treatment effect on the RPQ (primary outcome measure) and RPQ subscales (post-hoc analysis) for participants who completed the GAIN intervention based on an unadjusted and adjusted linear mixed model. Completers are defined as participants who received one or more group sessions and at least one individual session.
* Basic adjustment: Adjusted for age, sex, recruitment (ED/referred from GP), and previous mental health status (i.e. previous psychiatric diagnosis, yes/no)
**Full adjustment: Adjusted for basic adjustments and education level, functional somatic syndrome diagnosis prior to injury (yes/no), current psychological distress (SCL-8)
Abbreviations: RPQ=Rivermead Post-concussion symptoms Questionnaire.

Table S5. Difference in change between GAIN+EUC and EUC in RPQ total score from baseline to 3-month follow-up by predefined potential confounders (n=310)

Potential confounder	Level	Estimate	95% CI	p-value (a)
Sex				0.8990
	Female	-2.33	(-5.49 to 0.65)	
	Male	-2.58	(-6.86 to 1.48)	
Age				0.5050
	15 - 30 yrs	-3.59	(-7.44 to 0.05)	
	31 - 49 yrs	-1.08	(-4.89 to 2.50)	
	>=50 yrs	-2.71	(-7.04 to 1.50)	
Mental Health disorder				0.0236
	No previous mental health disorder	-3.96	(-7.31 to -0.94)	
	Previous mental health disorder	0.24	(-3.45 to 3.95)	
Education				0.1785
	Basic Education	2.85	(-4.44 to 9.85)	
	High School	-6.59	(-12.22 to -1.17)	
	Vocational Education	-0.31	(-5.27 to 4.77)	
	Higher Education	-2.79	(-6.13 to 0.26)	
	Other incl. missing	1.97	(-12.41 to 15.56)	
Previos functional disorder				0.9222
	No functional disorder	-2.46	(-5.64 to 0.52)	
	Functional disorder	-2.24	(-6.50 to 1.78)	
Current psychological distress				0.3597
	Quantile 1 (lowest)	-0.31	(-4.56 to 3.96)	
	Quantile 2	-3.07	(-7.39 to 0.89)	
	Quantile 3	-4.44	(-8.74 to -0.45)	
	Quantile 4 (highest)	-1.33	(-6.08 to 3.28)	
Recruitment				0.3438
	Emergency department	-1.14	(-5.18 to 2.75)	
	General practitioners	-3.03	(-6.26 to -0.02)	
Treatment Cluster				0.4341
	Horsens	-1.66	(-13.01 to 9.70)	
	Midt	-2.53	(-6.97 to 1.88)	
	Randers	-4.79	(-10.17 to 0.57)	
	Vest	0.44	(-3.87 to 4.79)	
	Aarhus	-3.63	(-7.44 to 0.18)	
Calendar time				0.0629
	2021 Aug-Nov	0.26	(-6.78 to 7.19)	
	2022 Feb-May	0.20	(-3.73 to 4.04)	
	2022 Aug-Nov	-4.37	(-7.73 to -1.11)	

Average treatment effects of RPQ at primary endpoint (3-months follow-up) subdivided by potential confounders in the fully adjusted model. All estimates were adjusted for calendar time, cluster, age, sex, recruitment, previous mental health status, education level, functional somatic syndrome diagnosis prior to injury, psychological distress.
(a) p-value for the likelihood ratio test of no treatment specific effects for each characteristic, i.e., there is post-hoc evidence of treatment-specific effect across previous mental health status.
