# Supplementary material for: Interdisciplinary intervention (GAIN) vs. enhanced usual care for reducing severe post-concussion symptoms in adults 2–4 months post-injury: a stepped wedge cluster randomised controlled trial
Source: eClinicalMedicine. 2026 Sep 22;100:104243. doi: 10.1016/j.eclinm.2026.104243 (PMC13672966; doi:10.1016/j.eclinm.2026.104243)
Supplement: Supplementary file 2 [file mmc2.pdf]

Næss-Schmidt *et al. Trials* (2022) 23:613
https://doi.org/10.1186/s13063-022-06572-7

Trials

STUDY PROTOCOL Open Access

Interdisciplinary intervention (GAIN) for adults with post-concussion symptoms: a study protocol for a stepped-wedge cluster randomised trial

Erhard Trillingsgaard Næss-Schmidt[1,2*], Mille Møller Thastum[1], Henriette Holm Stabel[1], Lene Odgaard[1], Asger Roer Pedersen[1,2], Charlotte Ulrikka Rask[2,3], Noah D. Silverberg[4], Andreas Schröder[5] and Jørgen Feldbæk Nielsen[1,2]

Abstract

Background: Persistent post-concussion symptoms (PCS) are associated with prolonged disability, reduced health-related quality of life and reduced workability. At present, no strong evidence for treatments for people with persistent PCS exists. Our research group developed a novel intervention, "Get going After concussIoN (GAIN)", that incorporates multiple evidence-based strategies including prescribed exercise, cognitive behavioural therapy, and gradual return to activity advice. In a previous randomised trial, GAIN provided in a hospital setting was effective in reducing symptoms in 15–30-year-olds with PCS 2–6 months post-injury. In the current study, we describe the protocol for a trial designed to test the effectiveness of GAIN in a larger municipality setting. Additionally, we test the intervention within a broader age group and evaluate a broader range of outcomes. The primary hypothesis is that participants allocated to enhanced usual care plus GAIN report a higher reduction in PCS 3 months post-intervention compared to participants allocated to enhanced usual care only.

Methods: The study is a stepped-wedge cluster-randomised trial with five clusters. The 8-week interdisciplinary GAIN program will be rolled out to clusters in 3-month intervals. Power calculation yield at least 180 participants to be enrolled. Primary outcome is mean change in PCS measured by the Rivermead Post-Concussion Symptoms Questionnaire from enrolment to 3 months after end of treatment. Secondary outcomes include participation in and satisfaction with everyday activities, labour market attachment and other behavioural measures. Self-reported outcomes are measured at baseline, by end of treatment and at 3, 6, and 18 months after end of treatment. Registry-based outcomes are measured up to 36 months after concussion.

Discussion: The trial will provide important information concerning the effectiveness of the GAIN intervention in a municipality setting. Furthermore, it will provide knowledge of possible barriers and facilitators that may be relevant for future implementation of GAIN in different settings.

*Correspondence: erhnae@rm.dk

[1] Hammel Neurorehabilitation Centre and University Research Clinic, Health, Aarhus University, Aarhus, Denmark
Full list of author information is available at the end of the article

© The Author(s) 2022. **Open Access** This article is licensed under a Creative Commons Attribution 4.0 International License, which permits use, sharing, adaptation, distribution and reproduction in any medium or format, as long as you give appropriate credit to the original author(s) and the source, provide a link to the Creative Commons licence, and indicate if changes were made. The images or other third party material in this article are included in the article's Creative Commons licence, unless indicated otherwise in a credit line to the material. If material is not included in the article's Creative Commons licence and your intended use is not permitted by statutory regulation or exceeds the permitted use, you will need to obtain permission directly from the copyright holder. To view a copy of this licence, visit http://creativecommons.org/licenses/by/4.0/. The Creative Commons Public Domain Dedication waiver (http://creativecommons.org/publicdomain/zero/1.0/) applies to the data made available in this article, unless otherwise stated in a credit line to the data.

Trial registration: The current GAIN trial is registered in ClinicalTrials.gov (study identifier: NCT04798885) on 20 October 2020.

Keywords: Brain concussion, Behavioural therapy, mTBI, Activities of daily living, Return to work, The Rivermead Post-Concussion Questionnaire

Administrative information

Note: the numbers in curly brackets in this protocol refer to SPIRIT checklist item numbers. The order of the items has been modified to group similar items (see http://www.equator-network.org/reporting-guidelines/spirit-2013-statement-defining-standard-protocol-items-for-clinical-trials/).

Title {1}	Interdisciplinary intervention (GAIN) for adults with post-concussion symptoms: a study protocol for a stepped-wedge cluster randomised trial
Trial registration {2a and 2b}.	ClinicalTrials.gov ID: NCT04798885. Registered on 12 March 2021
Protocol version {3}	18.05.2022 version 1
Funding {4}	The project is funded by Sygeforsikringen "danmark".
Author details {5a}	[1]Hammel Neurorehabilitation Centre and University Research Clinic, Health, Aarhus University, Denmark<br>[2]Department of Clinical medicine, AU<br>[3]Department of Child and Adolescent Psychiatry, Psychiatry, Aarhus University Hospital, Denmark<br>[4]Department of Psychology, University of British Columbia<br>[5]Research Clinic for Functional Disorders, Aarhus University Hospital, Aarhus, Denmark
Name and contact information for the trial sponsor {5b}	The project is sponsored by the foundation Sygeforsikringen "danmark", Palægade 5, 1261 København K
Role of sponsor {5c}	The sponsors have no involvement in managing the trial or in regard to collection, analysis and interpretation of data. Positive as well as negative outcome will be published in peer reviewed scientific journals.

Introduction

Background and rationale {6a}

Concussion, the mildest form of traumatic brain injury, is an important public health concern [1]. According to prospective studies, people reporting severe post-concussion symptoms (PCS) are associated with prolonged disability, reduced health-related quality of life and reduced workability [2–4]. Persistent PCS are associated with a high societal burden due to persistent impact on labour market attachment, and increased use of health care services and social welfare benefits [5]. It is estimated that persistent PCS such as headache, dizziness and sleep disturbances, are present in almost half of the affected persons 1-year post-injury [6].

Psychological factors are likely involved in the development of persistent PCS. In particular, negative illness perceptions worsen prognosis following concussion [7–9]. Furthermore, negative illness perceptions are associated with maladaptive illness behaviours such as avoiding activities, excessive rest, or the so-called all-or-nothing behaviour, i.e. oscillations between periods with very high or very low physical and mental activities [7, 10, 11]. Negative illness perceptions and maladaptive illness behaviours may act as symptom-perpetuating factors and reinforce PCS.

At present, no strong evidence for treatments for persistent PCS exists. However, recent systematic reviews and guidelines point toward principles from graded exercise therapy (GET), cognitive behavioural therapy (CBT) and a an interdisciplinary approach [12–15] may reduce symptoms or change illness behaviour and perception. These findings are in accordance with international guidelines for chronic pain management which also recommend CBT and GET [16, 17]. In line with this, three recent methodologically rigorous clinical trials demonstrated that an interdisciplinary intervention based on these principles were effective in reducing persistent PCS [14, 15, 18].

The intervention Get going After concussIoN (GAIN), was developed by the research group of the present study. GAIN was developed through a comprehensive process in a collaboration between people having had a concussion, therapists, experienced clinicians, and researchers [19]. The first GAIN trial (hereafter referred to as GAIN 1.0) tested the effect of GAIN delivered in a specialised hospital setting, among adolescents and young adults (15–30 years). In a randomised design, people having had a concussion receiving Enhanced Usual Care (EUC) plus GAIN (EUC+GAIN) showed a significantly larger reduction in PCS 3 months post-intervention compared to participants receiving EUC only. Furthermore, GAIN was demonstrated to be safe, feasible and associated with high participant satisfaction [19].

In recent years there has been an increased awareness of societal and personal consequences of persistent PCS in Denmark as well as internationally. Health

care professionals and social workers point to the gap in knowledge concerning the management of persistent PCS in the community setting, and they urgently advocate for evidence-based treatment [20]. Therefore, we find it relevant to test the effectiveness of GAIN in a second trial (hereafter referred to as GAIN 2.0) in a larger scale in 16 of the 19 municipalities in the Central Denmark Region, where the majority of people who experience persistent PCS are being cared for by physical therapist and occupational therapists, social workers and general practitioners (GPs). Additionally, the GAIN 2.0 study will test the intervention within a broader age group and evaluate whether GAIN promotes people's return to work and participation in everyday activities. Evidence from this study could facilitate wider uptake of GAIN, and thereby reduce personal and economic consequences of concussion.

Objectives {7}

The overall aim of GAIN 2.0 is to test the effectiveness of EUC + GAIN in a municipality setting. The effect is measured as changes in self-reported PCS, self-reported participation in and satisfaction with instrumental activities of daily living (IADL), and labour market attachment.

Primary hypothesis

Participants allocated to EUC + GAIN report a greater reduction in PCS 3 months post-intervention compared to participants allocated to EUC only.

Secondary hypotheses

1. Participants allocated to EUC + GAIN report a greater increase in participation in IADL 3 months post-intervention compared to participants allocated to EUC only measured by the Utrecht Scale for Evaluation of Rehabilitation-Participation (USER-P) [21] as sum scores on the following dimensions: more time spend on, less limitations experienced in, and more satisfaction with participation in IADL.
2. Participants allocated to EUC + GAIN have stronger labour market attachment 12 and 36 months post-concussion compared to participants allocated to EUC measured by sick leave, employment rate, and job stability.

Trial design {8}

GAIN 2.0 is embedded in a regional, population-based cohort study—hereafter referred to as "the epidemiologic study". GAIN 2.0 is a stepped-wedge cluster-randomised trial rolled out to clusters in 3-month intervals (Fig. 1). The trial consists of an 8-week interdisciplinary intervention programme (EUC + GAIN) against EUC only.

Methods: Participants, interventions and outcomes

Study setting {9}

GAIN 2.0 is carried out in Central Denmark Region (population 1.3 million) as a collaboration between Hammel Neurorehabilitation Centre and University Research Clinic (HNC) and 16 of the regions' 19 municipalities which are organised in five health clusters. GAIN is delivered at one or two specific locations in each of the five clusters.

GAIN 2.0 is embedded in an epidemiologic cohort study which includes people diagnosed with concussion at an emergency ward in Central Denmark Region and referred to the project by GP's. The cohort is followed by means of questionnaires and health registers. People with PCS $\geq$ 20 on the Rivermead Post-Concussion Symptoms Questionnaire (RPQ) are recruited for the present study from May 2021 to November 2022.

Eligibility criteria {10}

At enrolment, a clinical assessment of all participants is performed by medical doctors at the HNC. The clinical assessment consists of a neurological examination and

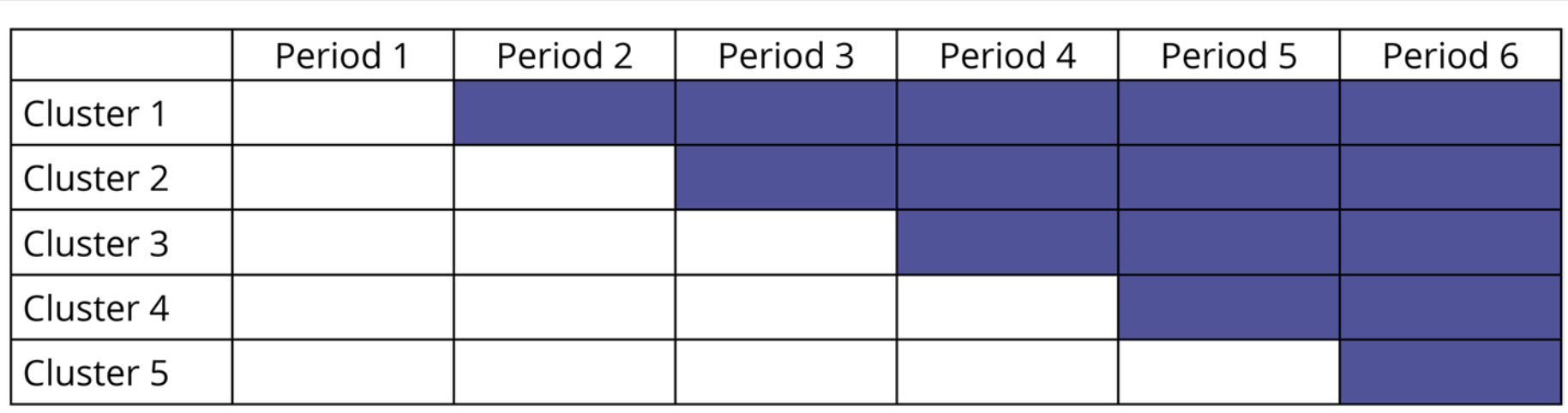

Fig. 1 The stepped wedge cluster randomised design. The stepped-wedge cluster randomised design with five clusters and six periods of each 3 months length. The white boxes illustrate the control condition (Enhanced Usual Care (EUC)) and the grey boxes intervention condition (EUC + Get Going After concussIoN (GAIN)). Recruitment, clinical assessment, and EUC take place 3 months period before each period illustrated in the figure. From power calculation, each box is estimated to involve a mean of 6 participants

a short, standardised medical history interview. Non-eligible participants are recommended to contact their GP for further advice if needed.

Participants are recruited based on the following inclusion criteria: (1) concussion caused by a head trauma according to the diagnostic criteria recommended by the Danish Consensus Report on Commotio Cerebri [22]. The criteria are based on recommendations by the WHO Task Force [2], but with the amendment, that there must have been a direct contact between the head and an object; (2) age 18 to 60 years at the time of the trauma; (3) a score of ≥ 20 on the RPQ within 1 week before enrolment in the study; (4) able to understand, speak and read Danish; (5) living in Central Denmark Region; and (6) identified from registers of the emergency departments or referred by GPs to GAIN 2.0 within 2–4 months after a concussion.

Exclusion criteria are (1) objective neurological findings and/or acute trauma CT scan indicating neurological disease or brain damage linked to the concussion, if performed; (2) previous concussion within the last 2 years with ongoing PCS at the time of the present concussion; (3) severe misuse of alcohol, prescription drugs and/or illegal drugs; (4) severe psychiatric co-morbidity (e.g. bipolar disorder, autism, psychotic disorder (life time) or severe neurological disease (e.g. multiple sclerosis) that impedes participation in the programme; and (5) inability to start the intervention within the maximum time for eligibility (4 months) plus the maximum possible waiting time from assessment to start of intervention (3 months), i.e. 7 months after concussion.

Who will take informed consent? {26a}

Prior to the clinical assessment and inclusion in the GAIN 2.0 programme, written informed consent is obtained from each participant.

Informed consent is based on both the written information on the GAIN 2.0 project, which is emailed to potential participants prior to the clinical assessment and verbal information provided through a video or telephone consultation a week before the clinical assessment by a medical doctor. In case of no prior video or telephone consultation, the verbal information is provided by the medical doctor just before the clinical assessment. The medical doctors are not engaged in performing neither EUC nor GAIN intervention.

Additional consent provisions for collection and use of participant data and biological specimens {26b}

The study does not involve biological specimens.

Interventions

Explanation for the choice of comparators {6b}

There is no strong evidence for any single intervention for people with persistent PCS. Supported by weak evidence [23], written or oral early information, reassurance and advice is a standard of care [24]. We consider it most ethically defensible to provide this standard of care (EUC) to all participants and compare the GAIN intervention to EUC only [15].

Intervention description {11a}

EUC + GAIN

Participants allocated to EUC + GAIN will receive the GAIN intervention in the municipality. GAIN is an 8-week, interdisciplinary intervention programme that aims to achieve changes in negative illness perceptions and maladaptive behaviours. The intervention includes psychoeducation based on principles from CBT, GET and gradual return to daily activities e.g. coping strategies, behavioural reactions to symptoms in everyday living, individual goal-setting, and physical exercise facilitating the increase of frequency, intensity and time [19]. Furthermore, a toolbox regarding advise on specific shoulder and neck exercises, sleep behaviour, ergonomics and relaxation training is provided if relevant in individual cases. In short, the programme is structured as (1) three structured group sessions of 2 h duration in the first, second and eighth week, led by a neuropsychologist, an occupational- and a physiotherapist which aims to give participants a general knowledge. Relatives are invited to the first and the second session. (2) Up to five semi-structured and tailored individual sessions of 30 min duration with an allocated therapist (either an occupational- or a physiotherapist) dispersed over the weeks between the second and last group session. Furthermore, the neuropsychologist provides weekly supervision to therapists throughout the intervention period to ensure quality and fidelity. Each individual session is performed either in person or video by choice of the participant, and homework is required between each session. The number of individual sessions is flexible (a maximum of five), and adjusted according to the participant's needs and goals. If neither a physical meeting nor video consultation was possible the individual session was arranged by telephone. The intervention is described in a manual which is available for the therapist involved in the intervention and will be released after the trial at the webpage of HNC.

Criteria for discontinuing or modifying allocated interventions {11b}

Participants are informed that they are free to withdraw their consent and reject further participation in GAIN

2.0 at any time. Previous results from the GAIN 1.0 trial showed no adverse effects of the GAIN intervention and therefore we do not expect any adverse effects of the intervention in GAIN 2.0 either. Short symptom deterioration may be expected because of exposure to e.g. GET and the intervention will be adjusted according to follow individual tolerance.

Strategies to improve adherence to interventions {11c}

To support adherence, GAIN incorporates personal goal-setting. Overall long-term goals are defined at the intervention start and short-term goals are then performed every week to be followed up in the next individual session.

Relevant concomitant care permitted or prohibited during the trial {11d}

All five health clusters begin with a 3 months control period where each municipality offers usual services for people with persistent PCS. After 3 months, the first health cluster is randomly selected to initiate the GAIN intervention. The municipalities of the remaining clusters continue to offer their usual services including potential existing intervention programmes addressing persistent PCS, until their health cluster is assigned to initiate the GAIN intervention and then GAIN takes over as the municipality services that are provided. A cluster will be assigned to initiate GAIN every third month to allow the 8-week intervention to be performed within 3 months regardless of holidays. Correspondingly, participants are enrolled over periods of 3 months before intervention start. This design is necessary to get sufficient participants to start up group sessions of the GAIN programme. Consequently, participants may have a waiting period of up to 3 months after enrolment before beginning the GAIN intervention. During this period, therapists in the municipalities are allowed to provide a maximum of two sessions of usual services such as advice regarding return to work and IADL. To avoid contamination, therapists educated in GAIN will not perform usual services for participants in the waiting period. At the end of the trial period, the GAIN intervention is provided in all five clusters. During the 8-week GAIN intervention participants are recommended to (but not demanded to) pause any other treatment for persistent PCS.

Provisions for post-trial care {30}

After the GAIN intervention, participants are free to continue or seek additional treatment for persistent PCS. Participants allocated to EUC are advised to seek further help from their GP if EUC is not sufficient.

Outcomes {12}

Primary outcome is mean change in PCS level as measured by the RPQ from baseline to 3 months after end of treatment. RPQ is a 16-item, self-reported scale measuring the severity of PCS including physical (e.g. headaches or vomiting), cognitive (e.g. poor concentration or forgetfulness) and emotional (e.g. feeling frustrated or depressed) symptoms, within the past 24 h compared to before the injury on a five-point scale (ranging from 0 “not experienced at all” to 4 “a severe problem”, range: 0–64; furthermore, as previously recommended, scores of 1 counts as 0 which we also apply) [25].

Secondary outcomes include participation in IADL at 3 months after end of treatment, measured as the sum scores in each of the three dimensions (‘time spend on daily activities’, ‘experienced limitations’, and ‘satisfaction with participation’) on the USER-P [21]. USER-P is a disease-nonspecific questionnaire that measures IADL such as work, voluntary work, education, family duties and responsibilities, leisure activities, transportation, communication, and social activities. Additional secondary outcomes include labour market attachment assessed by using data from the DREAM register [26] (i.e. incidence of long-term sick leave defined as public assistance benefits related to illness in more than three consecutive weeks within 12 and 36 months of concussion), proportion of employed participants (defined as receiving no public assistance benefits except from state education fund grants at 12 and 36 months after concussion), and degree of job stability (based on whether labour market contributions have been paid in the 1-month period before 12 and 36 months after concussion). Measures of treatment targets (i.e., mechanistic outcomes) include the Brief Illness Perception Questionnaire (B-IPQ) [27] and the Behavioural Response to Illness Questionnaire (BRIQ) [28]. Additional secondary outcomes are listed in additional files (see, Additional file 1).

All self-reported outcomes are measured at baseline (within 1 week before clinical examination), at end of treatment and at 3, 6 and 18 months after end of treatment. Additionally, RPQ, BRIQ and B-IPQ are measured four times during intervention in the EUC + GAIN group. Furthermore, DREAM register data will be extracted up to 36 months after concussion.

Participant timeline {13}

The time schedule for participants is illustrated in Fig. 2.

Sample size {14}

In GAIN 1.0, we found a mean difference in the improvement of 7.6 points between EUC + GAIN and EUC on the RPQ 3 months after end of treatment. In GAIN 2.0,

	EUC+GAIN	EUC
**Clinical assessment for eligibility, Enhanced usual care (EUC)** 2-7 months post-concussion	A, B	A, B
**Randomization** Within 2 weeks after clinical assessment	C	C
**GAIN intervention** Within 3 months after clinical assessment	D, E	
**End of treatment** 8 weeks after intervention start	E	E
**Follow-up period**	F	F
**3 months follow-up (Primary endpoint)** 3 months after end of treatment.	E	E
**Follow-up period**	F	F
**12 months follow-up** 12 months after end of treatment	E	E
**Follow-up period**	F	F
**18 months follow-up** 18 months after end of treatment	E	E

A	Participants are referred to the clinical assessment by GPs or invited through surveys among people identified from registers within 2-4 months post concussion. The clinical assessment is performed within 3 months of the invitation (a maximum of 7 months from intervention start) and includes a neurological examination, a short, standardized medical history interview and the baseline questionnaires to be answered within 1 week before the clinical assessment.
B	At the end of the clinical assessment, patients receive EUC see details in the text
C	Allocation is e-mailed to participants
D	The GAIN intervention is performed within 7 months after concussion (i.e. the maximum time of reference to clinical assessment (4 months) plus the maximum possible waiting time from assessment to start of intervention (3 months). Questionnaire data is collected four times during intervention.
E	Questionnaire data is collected three times during follow-up. For details see Additional file 1.
F	Usual care may be delivered between end of treatment and follow up periods by primary care physician, specialists and the municipalities.

Fig. 2 Timeline. Timing and characteristics of questionnaire and treatment elements delivered in each group

we hypothesize a difference in the improvement of 7 points between groups on the RPQ. Using the data from GAIN 1.0, the statistical power of the stepped-wedged design can be estimated. With the assumptions of a population mean RPQ change of 9.3 points of EUC + GAIN compared with EUC, measurement variance of 137, a variance between clusters of 0.97, a statistical significance level of 5%, and a statistical power of 80%, we will need 180 participants, i.e. six participants in each of the six clusters for every of six intervention periods. In the first GAIN study, we recruited 55 citizens each year in the age group between 15 and 30 years. By expanding the range of age and offering the intervention in five clusters covering 17 municipalities, we expect to recruit at least 180 participants in the project period.

Recruitment {15}

The research group, directing committee and one responsible key person from each cluster collaborate throughout the project to ensure the sustainability of recruitment and communication between clusters and their organisational levels. To facilitate progress in the study and ensure awareness of the study, information to GPs and collaborators will be continuously distributed through

newsletters and internal communication platforms. If the recruitment rate declines, information towards GP's and municipalities will be repeated and recruitment activities on social media will be increased.

Assignment of interventions: allocation

Sequence generation {16a}

A randomised schedule with time-points for start of intervention in each of the five clusters was computer-generated before trial start, by a statistician not involved in any other study procedures. The randomisation schedule is only accessible to the statistician.

Concealment mechanism {16b}

All clusters begin as EUC (control) groups. The five clusters are then one by one randomised to begin EUC + GAIN every third month. The statistician stores the result of the randomisation procedure. Three months before a new cluster start as an intervention group, the statistician informs the project coordinator of the next cluster. The project coordinator subsequently informs the key persons in the cluster and the data manager of the project.

Implementation {16c}

Participants are enrolled by the medical doctors after the clinical assessment at HNC. Within 2 weeks after the clinical assessment, the data manager registers the randomisation group of each participant and the participant subsequently receives an auto-generated e-mail with information on allocation.

Assignment of interventions: Blinding

Who will be blinded {17a}

The medical doctors assessing for eligibility are blinded to allocation of clusters. It is not possible to blind the therapists and the neuropsychologist performing GAIN in the municipalities. Only the project data manager has full access to the collected data and will not be involved in primary outcome analysis. The primary data analyser will be masked to whether a specific participant has received the intervention, but will not be masked during post hoc analyses.

Procedure for unblinding if needed {17b}

Not relevant in the current trial.

Data collection and management

Plans for assessment and collection of outcomes {18a}

Data is primarily collected through online surveys using standardised and validated questionnaires e.g. data on the primary outcome (RPQ) which has shown good validity and reliability [29].

Additional data on labour market attachment will be extracted from the DREAM database. Participants' satisfaction with GAIN will be measured by The Experience of Service Questionnaire [30]. Furthermore, individual interviews with 10–20 participants will be performed [31]. The semi-structured interviews will focus on the participants' experiences of the intervention, identify inconveniencies, and explore the possible active ingredients of GAIN. Finally, data from audio recordings of individual sessions and group sessions will be collected to check fidelity.

An overview of all measures and data sources is provided in additional files (see Additional file 1). All data collection and management are in compliance with Danish Data Protection Legislation and handling of data will be conducted according to general guidelines for encryption and anonymization (data handling number: 1–16-02–69-21).

Plans to promote participant retention and complete follow-up {18b}

Responses to questionnaires will be observed closely, and reminders are managed by the project data manager and a project assistant. In case of no or incomplete response to the questionnaires, participants will be contacted twice by e-mail, and lastly by telephone to collect as a minimum RPQ data by an interview. Non-responders will be included in the analyses of registry-based outcomes (see the "Outcomes {12}" section and Additional file 1).

Data management {19}

Study data are collected and managed using REDCap electronic data capture tools hosted by Aarhus University [32]. REDCap (Research Electronic Data Capture) is a secure, web-based software platform designed to support data capture for research studies, providing (1) an intuitive interface for validated data capture; (2) audit trails for tracking data manipulation and export procedures; (3) automated export procedures for seamless data downloads to common statistical packages; and (4) procedures for data integration and interoperability with external sources. All questionnaires are set up using REDCap validation features (e.g. range checks for data values). The project data manager administers REDCap access and check data quality within the REDCap system during the whole project.

Confidentiality {27}

All data collection and storage are managed through surveys and registration forms in REDCap, except for interviews which are stored in a safety box. During the whole project period, the data manager regularly removes REDcap access for users who are no longer affiliated to the

project and ensures that users are only granted necessary user rights (e.g. therapist will not have access to questionnaire data entered by the participants). Additionally, therapists will only be granted rights to see the participant's personal registration number during intervention while the participants receive GAIN.

Data to be merged with register-based data will be extracted from REDCap and thereafter immediately uploaded to the research computers at Statistics Denmark using the Statistics Denmark encrypted upload function.

Plans for collection, laboratory evaluation and storage of biological specimens for genetic or molecular analysis in this trial/future use {33}
The project does not involve biological specimens.

Statistical methods
Statistical methods for primary and secondary outcomes {20a}
Analyses are handled on an intention-to-treat basis. The primary outcome, RPQ, is analysed by using linear mixed-effects models with fixed effect of intervention arm and time (baseline, end of treatment, and 3 months after end of treatment) in interaction and random effects of clusters and subjects within clusters. Similarly, to evaluate clinical significance, we calculate the relative risk in each intervention arm of having an RPQ total score ≥ 20 at 3 months after end of treatment as well as the number needed to treat for prevention of one additional subject with RPQ total score ≥ 20. The secondary outcomes are analysed using linear mixed models similar to those described for the primary outcome using the secondary outcomes as dependent variables instead of RPQ. DREAM data will be analysed with time-to-event analysis and logistic regression.

Interim analyses {21b}
The project does not involve interim analysis because the time schedule and design are fixed and end when the fifth cluster finishes GAIN at the end of the project period.

Methods for additional analyses (e.g. subgroup analyses) {20b}
Both primary and secondary outcomes are analysed in crude and adjusted models including age, sex, previous mental health issues (no/yes), and recruitment (GP/hospital registry in Central Denmark Region).

Methods in analysis to handle protocol non-adherence and any statistical methods to handle missing data {20c}
Additionally, per-protocol analysis for completers defined as participants who received one or more group sessions and at least one individual session is performed. No further methods will be used to handle missing data.

Plans to give access to the full protocol, participant level-data and statistical code {31c}
The data utilised in the current study is defined as sensitive personal data. The data cannot be shared publicly due to existing data protection laws in Denmark imposed by the Danish Data Protection Agency. The access may be granted on anonymised data and case-by-case basis by approval from the project group who has the legal responsibility as owner and data manager. Access will be granted to the extent permissible by the General Data Protection Regulation and the Danish Data Protection Act. In this case, the principal investigator (Jørgen Feldbæk Nielsen, e-mail: joerniel@rm.dk) will make data available for the investigator through his affiliated research institution in Denmark with approved authority to access the data. The General Data Protection Regulation and the Danish Data Protection Act and regulations prohibit all other forms of data sharing.

Oversight and monitoring
Composition of the coordinating centre and trial steering committee {5d}
The project organisation is composed by a directing committee meeting every third month (including the principal investigators PI, JN, and EN and three representatives of the Central Denmark Region), five municipal leaders each responsible of one of the five clusters, researchers (research group and the PIs), a research support group (including project assistant and communication worker) and an international expert panel. See Additional file 2 for an overview.

The directing committee and municipal cluster leaders are responsible for the risk management throughout the project and for ensuring the successful delivery of GAIN 2.0 in the municipalities. The research group will perform the recruitment, the clinical assessment, the education in GAIN for therapists in each of the clusters, and the daily data management; they meet once every month. The municipal leaders (leaders of each of the five clusters) are responsible for providing therapists and local facilities for delivering GAIN to participants. Challenges and reflections of the process are shared with the international expert panel on meetings scheduled every fifth month.

Composition of the data monitoring committee, its role and reporting structure {21a}
Data monitoring committee is not included in the trial as sponsors are not involved in the study design, data collection or analysis and data are collected using public administered systems (REDCap and Statistics Denmark).

Adverse event reporting and harms {22}

Potential adverse events may actively be addressed through the survey after end of treatment and at 3 months end of treatment or passively through the intervention period if experienced by the therapists. Adverse events during the intervention are additionally registered by the municipal therapists.

Frequency and plans for auditing trial conduct {23}

Digital voice recordings are performed in group sessions and individual sessions throughout the trial to assess fidelity and adherence to the study protocol after obtaining participants' written consent for the recordings. After completion of the trial, 10% of the recordings are randomly selected from the group sessions and the individual sessions and checked by independent assessors with regard to adherence to the GAIN protocol. Furthermore, adherence to the treatment manual is monitored by the two neuropsychologists who provide weekly supervision to therapists delivering GAIN through out the study.

Plans for communicating important protocol amendments to relevant parties (e.g. trial participants, ethical committees) {25}

Any amendments to the protocol having a possible impact on outcome are registered to relevant registries or regulators, i.e. clinical trials, ethics committee in the Central Denmark Region, and record of processing activities including the General Data Protection Regulation. For details on the approved main protocol for the ethics committee; see Additional files 3 and 4.

Dissemination plans {31a}

The scientific dissemination will be performed by publishing results in international peer-reviewed scientific journals. The intention is to publish positive as well as negative or inconclusive results that will add to the scientific knowledge concerning the treatment of people with persistent PCS. Furthermore, results will be presented at national and international conferences. By the end of the study period, we will host a final seminar to enable the transfer of knowledge to municipalities nationally and patient organisations. In collaboration with the press offices at HNC and in the municipalities, we plan to execute a media strategy, including dissemination of results through social media, webpages, press releases, and feature articles. Furthermore, we aim at publishing popular featured articles in relevant Danish professional magazines. The aim is to inform the public, including people having had a concussion and relatives, health care professionals (e.g. therapists, medical doctors and other medical or social welfare staff), and to reach out for the policy makers outside the participating region and municipalities. In all parts of communication, dissemination and outreach, "Sygeforsikringen danmark" will be acknowledged and mentioned as a funder of the trial.

Discussion

At present, a limited number of people with persistent PCS in Denmark are offered treatment and the available interventions are very heterogenic. There is an increased consensus that persistent PCS is best understood in terms of a multifactorial bio-psycho-social model [7] but standardised interdisciplinary interventions are lacking. The present study tests the effectiveness of the originally hospital-based GAIN intervention developed by Thastum et al. [15] in a municipality setting. The study involves a realistic and pragmatic setup of the intervention close to participants' homes.

The present study has some limitations. First, blinding of the clinicians providing the intervention is not possible. This is a general and well-known problem in trials of behavioural interventions. Another challenge is to ensure fidelity to the treatment manual in a new and larger group of therapists. To ensure fidelity, an educational programme in GAIN to train new therapists was developed and piloted in a municipality outside the Central Denmark Region before the study commenced. Furthermore, two neuropsychologists with special knowledge of concussion and persistent PCS, cognitive behavioural therapy, and graded return to activities, are in charge of all group sessions and provide weekly supervision to therapists throughout the intervention period to ensure quality and fidelity. One of the neuropsychologists (MMT) was directly involved in the development of GAIN and was part of the interdisciplinary intervention team in GAIN 1.0. Another limitation is, that the five clusters (consisting of the 17 municipalities) participating in the trial have different approaches to usual care for people with persistent PCS, and some municipalities may offer more to participants allocated to EUC than others. This may reduce the estimated treatment effect of GAIN. Further selection bias may be an issue if proper randomisation of the five health clusters is not achieved. This will however be accounted for in the data analysis. Expectation bias may also affect both the intervention group and the control group in respectively positive and negative directions. The clinical assessment and advice at baseline may however partly adjust for the negative effect in the control group.

Finally, as the group sessions are a vital part in the GAIN programme, it is necessary to accept a

recruitment period of 3 months to be able to include a group of four to nine persons in each intervention group resulting in a waiting period of up to 3 months before beginning the intervention in the EUC+GAIN group. Therefore, participants in the EUC-only group may on average receive usual care earlier after injury than the EUC+GAIN group receive the GAIN intervention which could reduce the estimated effect of the GAIN programme.

In contrast to the hospital-based intervention in GAIN 1.0, a strength of GAIN 2.0 is that the intervention is delivered in a municipality setting close to the home of possible participants, and it is delivered by therapists employed in the municipalities where this population is normally treated. The municipalities may be a more feasible intervention setting in the future, demanding less transportation for participants and enabling a closer collaboration between therapists and social services in the municipalities.

Furthermore, in GAIN 2.0, the upper limit of 60 years is broader than the upper limit of 30 years of age applied in GAIN 1.0, and the result may therefore be more generalizable.

Another strength is the use of reliable and valid outcome measures based on self-reported surveys not administered by persons involved in the intervention. Moreover, predefined outcomes and trial registration before the beginning of the trial minimise the risk of bias.

A final strength may be the holistic and empowering nature of the GAIN programme in contrast to single symptom or passive treatment strategies.

In conclusion, this trial will provide important information on the interdisciplinary developed GAIN programme and its effectiveness in a municipality setting. If the programme is successful, it will improve and guide future directions for early treatment of people with persistent PCS facilitate wider uptake of GAIN, and thereby reduce personal and economic consequences of concussion.

Trial status

This is the first version of the protocol dated 30.04.2022. Enrolment of participants began in May 2021 and recruitment will end by November 2022.

Abbreviations

PCS: Post-concussion symptoms; GAIN: Get going After concussIoN; GET: Graded exercise therapy; CBT: Cognitive behavioural therapy; EUC: Enhanced Usual Care; EUC+GAIN: Enhanced Usual Care plus GAIN; GP: General practitioners; IADL: Instrumental activities of daily living; USER-P: Utrecht Scale for Evaluation of Rehabilitation-Participation; HNC: Hammel Neurorehabilitation Centre and University Research Clinic; RPQ: Rivermead Post-Concussion Symptoms Questionnaire; BIPQ: Brief Illness Perception Questionnaire; BRIQ: Behavioural Response to Illness Questionnaire.

Supplementary Information

The online version contains supplementary material available at https://doi.org/10.1186/s13063-022-06572-7.

Additional file 1. Outcome measures.

Additional file 2. Organisation.

Additional file 3.

Additional file 4.

Additional file 5.

Additional file 6. Samtykke GAIN 2.0.

Acknowledgements

We thank Toril Skandsen and Hana Malá Rytter for usefull discussions.

Authors' contributions {31b}

JN and ETN were the Chief Investigators and led the proposal and protocol development. All authors were engaged in the development of the design of the study. ETN performed the first draft of the protocol article. ARP provided statistical advice. All authors have read and critically appraised the manuscript several times and approved the final version.

Funding {4}

The project was funded by Sygeforsikringen "danmark". The funder was not involved in the design of the study; collection, analysis, or interpretation of data; and in the writing of the manuscript.

Availability of data and materials {29}

The data in the current study is defined as sensitive personal data. The data cannot be shared publicly due to existing data protection laws in Denmark imposed by the Danish Data Protection Agency. The access may be granted on anonymised data and a case-by-case basis by approval from the project group (PIs and research group). See {31C} for details.

Declarations

Ethics approval and consent to participate {24}

The project will be carried out in accordance with the rules of the Helsinki Declaration II. Written and verbal information of the study will be given to participants before obtaining written informed consent. Also, participants will be informed that they are able to decline participation in the project at any time. Approval has been obtained from the National Committee on Health Research Ethics: 1–16-02–69-21.

Consent for publication {32}

Consent forms can be found in Additional files 5 (participation in the RCT) and 6 (participation in the interviews in the intervention group).

Competing interests {28}

The authors declare that they have no competing interests.

Author details

[1]Hammel Neurorehabilitation Centre and University Research Clinic, Health, Aarhus University, Aarhus, Denmark. [2]Department of Clinical Medicine, AU, Aarhus, Denmark. [3]Department of Child and Adolescent Psychiatry, Psychiatry, Aarhus University Hospital, Aarhus, Denmark. [4]Department of Psychology, University of British Columbia, Vancouver, Canada. [5]Research Clinic for Functional Disorders, Aarhus University Hospital, Aarhus, Denmark.

Received: 29 May 2022 Accepted: 20 July 2022
Published online: 29 July 2022

References

1. Carroll L, Cassidy JD, Peloso P, Borg J, von Holst H, Holm L, et al. Prognosis for mild traumatic brain injury: results of the who collaborating centre task force on mild traumatic brain injury. J Rehabil Med. 2004;36:84–105.
2. Cassidy JD, Carroll L, Peloso P, Borg J, x000F, rgen, et al. Incidence, risk factors and prevention of mild traumatic brain injury: results of the who collaborating centre task force on mild traumatic brain injury. J Rehab Med. 2004;36:28–60.
3. Graff HJ, Siersma V, Møller A, Kragstrup J, Andersen LL, Egerod I, et al. Labour market attachment after mild traumatic brain injury: Nationwide cohort study with 5-year register follow-up in Denmark. BMJ Open. 2019;9:e026104.
4. Ponsford J, Cameron P, Fitzgerald M, Grant M, Mikocka-Walus A, Schönberger M. Predictors of postconcussive symptoms 3 months after mild traumatic brain injury. Neuropsychology. 2012;26(3):304–13.
5. Graff HJ, Siersma V, Møller A, Kragstrup J, Andersen LL, Egerod I, et al. Five-year Trends in Marital Stability, Academic Achievement, and Socioeconomic Indicators After Concussion: A National Register Study. J Head Trauma Rehabil. 2020;35(2):E86–E94.
6. Theadom A, Parag V, Dowell T, McPherson K, Starkey N, Barker-Collo S, et al. Persistent problems 1 year after mild traumatic brain injury: a longitudinal population study in New Zealand. Br J Gen Pract. 2016;66(642):e16–23.
7. Hou R, Moss-Morris R, Peveler R, Mogg K, Bradley BP, Belli A. When a minor head injury results in enduring symptoms: a prospective investigation of risk factors for postconcussional syndrome after mild traumatic brain injury. J Neurol Neurosurg Psychiatry. 2012;83(2):217–23.
8. Snell DL, Surgenor LJ, Hay-Smith EJC, Siegert RJ. A systematic review of psychological treatments for mild traumatic brain injury: an update on the evidence. J Clin Exp Neuropsychol. 2009;31(1):20–38.
9. Patel N, Rao VA, Heilman-Espinoza ER, Lai R, Quesada RA, Flint AC. Simple and reliable determination of the modified rankin scale score in neurosurgical and neurological patients: The mRS-9Q. Neurosurgery. 2012;71(5):971–5.
10. Potter S, Brown RG. Cognitive behavioural therapy and persistent post-concussional symptoms: Integrating conceptual issues and practical aspects in treatment. Neuropsychol Rehabil. 2012;22(1):1–25.
11. McCrory P, Meeuwisse W, Dvořák J, Aubry M, Bailes J, Broglio S, et al. Consensus statement on concussion in sport—the 5th international conference on concussion in sport held in Berlin, October 2016. Br J Sports Med. 2017;51(11):838–47.
12. Marshall S, Bayley M, McCullagh S, Velikonja D, Berrigan L, Ouchterlony D, et al. Updated clinical practice guidelines for concussion/mild traumatic brain injury and persistent symptoms. Brain Inj. 2015;29(6):688–700.
13. Bergersen K, Halvorsen JØ, Tryti EA, Taylor SI, Olsen A. A systematic literature review of psychotherapeutic treatment of prolonged symptoms after mild traumatic brain injury. Brain Inj. 2017;31(3):279–89.
14. Rytter HM, Westenbaek K, Henriksen H, Christiansen P, Humle F. Specialized interdisciplinary rehabilitation reduces persistent post-concussive symptoms: a randomized clinical trial. Brain Inj. 2019;33(3):266–81.
15. Thastum MM, Rask CU, Næss-Schmidt ET, Tuborgh A, Jensen JS, Svendsen SW, et al. Novel interdisciplinary intervention, GAIN, vs. enhanced usual care to reduce high levels of post-concussion symptoms in adolescents and young adults 2–6 months post-injury: A randomised trial. EClinicalMedicine. 2019;17:2019–20.
16. Meeus M, Nijs J, Wilgen PV, Noten S, Goubert D, Huijnen I. Moving on to Movement in Patients with Chronic Joint Pain. international association for the society study of pain. 2016; Vol XXIV; Marts(1).
17. Skelly AC, Chou R, Dettori JR, Turner JA, Friedly JL, Rundell SD, et al. Noninvasive Nonpharmacological Treatment for Chronic Pain: A Systematic Review Update. AHRQ Comparative Effectiveness Reviews. 2020. Apr. Report No.: 20-EHC009
18. Silverberg ND, Cairncross M, Brasher PMA, Vranceanu AM, Snell DL, Yeates KO, et al. Feasibility of Concussion Rehabilitation Approaches Tailored to Psychological Coping Styles: A Randomized Controlled Trial. Arch Phys Med Rehabil. 2021;S0003-9993(21)01732–9.
19. Thastum MM, Rask CU, Naess-Schmidt ET, Jensen JS, Frederiksen O-V, Tuborgh A, et al. Design of an early intervention for persistent post-concussion symptoms in adolescents and young adults: a feasibility study. NeuroRehabilitation. 2018;43(2):155–67.
20. Schröder A, Thastum MM, Næss-Schmidt ET, Rask CU, Nilesen JF. Hvordan kan vi skabe optimale betingelser for at hjernen kan hele efter en hjernerystelse? Ugeskrift for læger. 2020;182(2).
21. Post MWM, Van Der Zee CH, Hennink J, Schafrat CG, Visser-Meily JMA, Van Berlekom SB. Validity of the utrecht scale for evaluation of rehabilitation-participation. Disabil Rehabil. 2012;34(6):478–85.
22. Pinner M, Børgesen SE, Jensen R, Birket-Smith M, Gade A, Riis J. Konsensusrapport om commotio cerebri (hjernerystelse) og det postcommotionelle syndrom. 2002.
23. Rytter HM, Graff HJ, Henriksen HK, Aaen N, Hartvigsen J, Hoegh M, et al. Nonpharmacological treatment of persistent postconcussion symptoms in adults: a systematic review and meta-analysis and guideline recommendation. JAMA Netw Open. 2021;4(11):e2132221.
24. Silverberg ND, Iaccarino MA, Panenka WJ, Iverson GL, McCulloch KL, Dams-O'connor K, et al. Management of concussion and mild traumatic brain injury: a synthesis of practice guidelines. Arch Phys Med Rehabil. 2020;101:382–93.
25. King NS, Crawford S, Wenden FJ, Moss NEG, Wade DT. The rivermead post concussion symptoms questionnaire: a measure of symptoms commonly experienced after head injury and its reliability. J Neurol. 1995;242(9):587–92.
26. Hjollund NH, Larsen FB, Andersen JH. Register-based follow-up of social benefits and other transfer payments: accuracy and degree of completeness in a Danish interdepartmental administrative database compared with a population-based survey. Scand J Public Health. 2007;35(5):497–502.
27. Broadbent E, Petrie KJ, Main J, Weinman J. The brief illness perception questionnaire. J Psychosom Res. 2006;60(6):631–7.
28. Spence M, Moss-Morris R, Chalder T. The Behavioural Responses to Illness Questionnaire (BRIQ): a new predictive measure of medically unexplained symptoms following acute infection. Psychol Med. 2005;35(4):583–93.
29. Eyres S, Carey A, Gilworth G, Neumann V, Tennant A. Construct validity and reliability of the rivermead post-concussion symptoms questionnaire. Clin Rehabil. 2005;19(8):878–87.
30. Brown A, Ford T, Deighton J, Wolpert M. Satisfaction in child and adolescent mental health services: Translating users' feedback into measurement. Adm Policy Ment Health Ment Health Serv Res. 2014;41(4):434–46.
31. Sandelowski M. Combining qualitative and quantitative sampling, data collection, and analysis techniques in mixed-method studies. Res Nurs Health. 2000;23(3):246–55.
32. Harris PA, Taylor R, Minor BL, Elliott V, Fernandez M, O'Neal L, et al. The REDCap consortium: Building an international community of software platform partners. J Biomed Inform. 2019;95: 103208.

Publisher's Note

Springer Nature remains neutral with regard to jurisdictional claims in published maps and institutional affiliations.

Ready to submit your research? Choose BMC and benefit from:

- fast, convenient online submission
- thorough peer review by experienced researchers in your field
- rapid publication on acceptance
- support for research data, including large and complex data types
- gold Open Access which fosters wider collaboration and increased citations
- maximum visibility for your research: over 100M website views per year

At BMC, research is always in progress.

Learn more biomedcentral.com/submissions

BMC
